# From Multi-Modal Property Dataset to Robot-Centric Conceptual Knowledge About Household Objects

**DOI:** 10.3389/frobt.2021.476084

**Published:** 2021-04-15

**Authors:** Madhura Thosar, Christian A. Mueller, Georg Jäger, Johannes Schleiss, Narender Pulugu, Ravi Mallikarjun Chennaboina, Sai Vivek Rao Jeevangekar, Andreas Birk, Max Pfingsthorn, Sebastian Zug

**Affiliations:** ^1^Faculty of Computer Science, Institute for Intelligent Cooperating Systems, Otto-von-Guericke University Magdeburg, Magdeburg, Germany; ^2^Robotics, Computer Science & Electrical Engineering Department, Jacobs University, Bremen, Germany; ^3^Institute for Computer Science, Technische Universität Bergakademie Freiberg, Freiberg, Germany; ^4^OFFIS Institute for Information Technology, Oldenburg, Germany

**Keywords:** conceptual knowledge, robot-centric knowledge, multi-modal dataset, knowledge acquisition, substitute selection

## Abstract

Conceptual knowledge about objects is essential for humans, as well as for animals, to interact with their environment. On this basis, the objects can be understood as tools, a selection process can be implemented and their usage can be planned in order to achieve a specific goal. The conceptual knowledge, in this case, is primarily concerned about the physical properties and functional properties observed in the objects. Similarly tool-use applications in robotics require such conceptual knowledge about objects for *substitute selection* among other purposes. State-of-the-art methods employ a top-down approach where hand-crafted symbolic knowledge, which is defined from a human perspective, is grounded into sensory data afterwards. However, due to different sensing and acting capabilities of robots, a robot's conceptual understanding of objects (e.g., light/heavy) will vary and therefore should be generated from the robot's perspective entirely, which entails robot-centric conceptual knowledge about objects. A similar bottom-up argument has been put forth in cognitive science that humans and animals alike develop conceptual understanding of objects based on their own perceptual experiences with objects. With this goal in mind, we propose an extensible property estimation framework which consists of estimations methods to obtain the quantitative measurements of physical properties (rigidity, weight, etc.) and functional properties (containment, support, etc.) from household objects. This property estimation forms the basis for our second contribution: Generation of robot-centric conceptual knowledge. Our approach employs unsupervised clustering methods to transform numerical property data into symbols, and Bivariate Joint Frequency Distributions and Sample Proportion to generate conceptual knowledge about objects using the robot-centric symbols. A preliminary implementation of the proposed framework is employed to acquire a dataset comprising six *physical* and four *functional* properties of 110 household objects. This Robot-Centric dataSet (RoCS) is used to evaluate the framework regarding the property estimation methods and the semantics of the considered properties within the dataset. Furthermore, the dataset includes the derived robot-centric conceptual knowledge using the proposed framework. The application of the conceptual knowledge about objects is then evaluated by examining its usefulness in a tool substitution scenario.

## 1. Introduction

**Figure 1:**
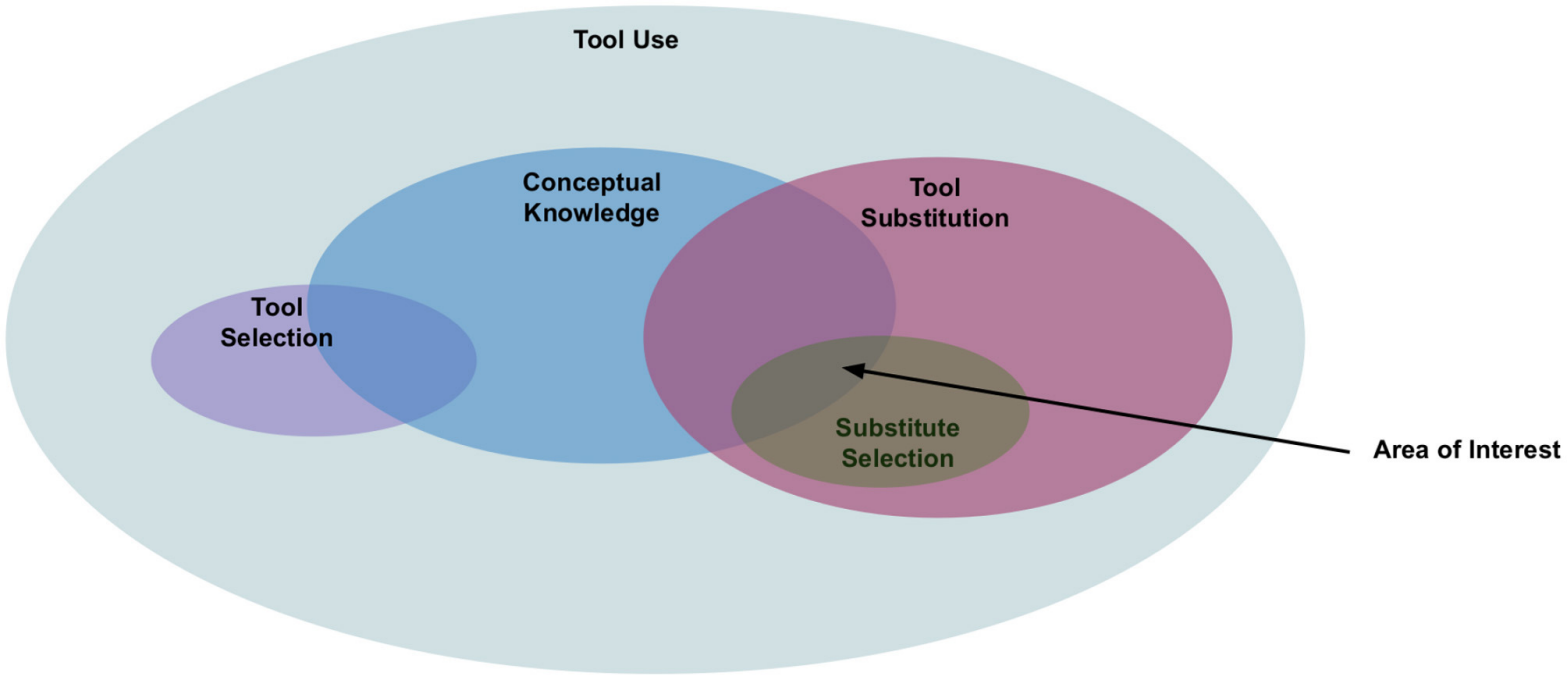
The figure shows our primary area of interest within the domain of tool-use. Conceptual knowledge is desirable in tool use, however our focus is on generating conceptual knowledge required for substitute selection. The figure also illustrates the positioning of substitute selection within tool-use. While tool-use also consists of areas such as grasping, planning, manipulation, validation etc. we have left them out for the sake of clarity. Besides our primary area of interest, our intent is to distinguish it from tool-use or tool selection. Note that in tool selection a robot does not have any prior knowledge of what is tool is appropriate in a given task whereas in substitute selection, the robot does have such prior knowledge.

Humans have become extremely sophisticated in their use of tools compare to their animal counterparts. The sophistication pertaining to tool-use in humans involves not just the dexterity in manipulating a tool, but also the diversity in tool exploitation (Gavin et al., 2013). The ability to exploit the tools has enabled humans to adapt and thus exert control over an uncertain environment, especially when they are faced with unfavorable situations. Given how vital the tool-use ability is, robotics researchers have been developing approaches to enable a robot to use tools in various tasks (Stoytchev, 2007; Brown and Sammut, 2012; Stückler and Behnke, 2014; Takahashi et al., 2014; Li and Fritz, 2015; Tikhanoff et al., 2015; Wicaksono and Sammut, 2018; Toussaint et al., 2019). While these approaches focus on learning tool-use behavior, our primary interest is in a question: what if the required tool is missing, given that robot has prior knowledge about what tool is required in the task (see Figure 1)?

Consider a scenario where a robot is performing a task and has to select between a stone and a plastic bottle for a hammering purpose. One way to reach a decision is to interact with each object, perform the action and determine its suitability on the basis of a desired outcome. However, in the real world there are many objects to select from, such (individual) interactions may not be desirable for completing the task in a reasonable amount of time as it will be time consuming to determine a suitability of individual objects. If the hands-on substitute selection is undesirable, then what is the alternative selection strategy?

In order to select a plausible substitute for a missing tool, the substitute needs to be similar to the missing tool in some way without having to interact with it. The question is what is needed to determine the similarity. In the literature on substitute selection, typically a substitute for a missing tool is determined by means of knowledge about object, and the knowledge-driven similarity between a missing tool prototype and a potential substitute. Such knowledge about objects varies in its contents and form across the literature: metric data about position, orientation, size, and symbolic knowledge about hand-picked relations such as *similar-to* and *capable-of* extracted from ConceptNet (Bansal et al., 2020); visual and physical understanding of multi-object interactions demonstrated by humans (Xie et al., 2019); matching similarity of shapes of point clouds and materials based on the spectrometer data using dual neural network (Shrivatsav et al., 2019); metric data about size, shape and grasp, as well as a human estimate of an affordance score for task + mass (Abelha and Guerin, 2017); attributes and affordances of objects are hand-coded using a logic-based notation, and a multidimensional conceptual space of features such as shape and color intensity (Mustafa et al., 2016); hand-coded models of known tools in terms of superquadrics and relationships among them (Abelha et al., 2016); potential candidates extracted from WordNet and ConceptNet if they share the same parent with a missing tool for predetermined relations: *has-property, capable-of*, and *used-for* (Boteanu et al., 2016); hand-coded object-action relations (Agostini et al., 2015); as well as hand-coded knowledge about inheritance and equivalence relations among objects and affordances (Awaad et al., 2014). While for tool selection, metric data of certain properties are primarily considered, for substitute selection, symbolic knowledge about the object category or class is considered. In such cases, either the proposed approaches use existing common sense knowledge bases such as WordNet, ConceptNet or knowledge is hand-coded. Regardless of the use of existing knowledge bases or hand-coded relational knowledge, the required knowledge is generally carefully selected for a given task.

It is postulated in the literature on tool-use in animals (Baber, 2003, Chapter 1) that “*a non-invasive tool selection in humans or animals alike is facilitated by conceptual knowledge about objects, especially, knowledge about their physical and functional properties and relationship between them*.” For instance, knowledge about what physical properties of a hammer enable the hammering action can facilitate the decision between a stone and a plastic bottle as a substitute. Conceptual knowledge about objects, in this case, is considered as a representation of objects in terms of its physical and functional properties generalized over our observations and daily interactions with them (Baber, 2003). Therefore, based on our observations and interactions with various instances of a cup, a conceptual knowledge of a *cup* may for example consist of an object that has a handle, is hollow and can contain liquid.

Like humans, such conceptual knowledge about objects is desired in robot systems (from household to industrial robotics) in order to efficiently perform tasks such as tool selection and substitute selection, where selection is driven by the knowledge about various (physical and functional) properties observed in the objects (Stoytchev, 2007; Brown and Sammut, 2013).

In this article, we present an approach to acquire relevant sensory data, estimate metrics of object (physical and functional) properties based on the data, and generate conceptual knowledge about objects in a bottom-up data-driven manner.

### 1.1. Building Blocks for Robot-Centric Conceptual Knowledge

In order to acquire the proposed conceptual knowledge about objects (e.g., in a household environment), the following questions need to be answered:

- What kind of knowledge constitutes conceptual knowledge about objects?
- How can conceptual knowledge about objects be acquired?
- How can the acquired knowledge be represented?

These questions forms the primary building blocks of our work, namely: *Conceptual Knowledge, Knowledge Representation*, and *Robot-Centric Knowledge*. In this section, we address how the building blocks are realized in this work.

#### 1.1.1. Conceptual Knowledge

Humans tend to express an object in linguistic form by giving it a label such as a mug (Rand, 1990). However, for humans, a mug is not merely a label, but rather it represents a concept that has properties such as rigid, hollow, cylindrical, ability to contain liquids, made up of ceramic material and also has a primary function, for instance, holds liquid (Hodges et al., 1999).

But is knowing merely “whether a cup is rigid or not” enough? Consider, for instance, a choice between a cup and a stone as a substitute for a hammer. While both the objects are rigid, we have *general knowledge* that a stone is *usually more rigid* than a cup and quite possibly as rigid as a hammer. As a result, we will choose the stone over the cup for hammering. Another example is the choice between a mobile phone and a plate as a substitute for a tray to carry a drink. Since both the objects are flat, they should be viable substitutes. However, since we *know* that a plate is *usually larger in size than* a mobile phone, and a plate is *closer to* a tray *in size* than a mobile phone is, we will vote for the plate. There are two pieces of information worth noticing: Firstly, our knowledge about properties of objects is *generalized, relative, subjective*, and *qualitative*, and secondly, the selected substitutes are not necessarily visually similar to the missing tools but are rather qualitatively similar.

We have based our approach toward conceptual knowledge about objects, which consists of qualitative knowledge about their properties, on the way humans form a concept around objects and its properties The properties are divided into *physical* (see section 2.2) and *functional* properties (see section 2.3). The physical properties describe the physicality of objects (*rigidity, weight, hollowness, roughness, flatness, size*) while the functional properties ascribe the (functional) abilities or affordances to the objects (*containment, blockage, support, movability*). The functional properties are derived from the theory of image schema (Gärdenfors, 1987) proposed in cognitive linguistics (see section 2.3).

#### 1.1.2. Robot-Centric Knowledge

The primary motivation for pursuing a robot-centric aspect stems from the research on cognitive aspects of tool use in humans and animals. Especially the theory that tool selection is a *first-person-perspective* activity which is driven by a relationship between the *user's own conceptual knowledge* about a tool and their ability to use that tool (Baber, 2003). We noted earlier that one of the aspects of conceptual knowledge that needs to be expressed is subjective knowledge. It has been argued in the cognitive science studies on concept formation that conceptual knowledge of an object is *grounded* in an individual's multi-modal perceptual experiences with various objects (Feldman and Narayanan, 2004; Gallese and Lakoff, 2005; Louwerse and Jeuniaux, 2010). This suggests that a conceptual understanding of any object may differ from person to person. This also holds true for robots as in general, as robots come in a multitude of perception and manipulation configurations. As a consequence, the individual perception and manipulation of the world similarly varies from robot to robot. Therefore, knowledge acquired about an object by a KUKA KR1000 Titan (maximum payload of 1,300 kg, 3.6 m reach), for example, will not be the same as knowledge acquired by a Universal Robot UR3 (maximum payload of 3 kg, 0.5 m reach).

**Figure 2:**
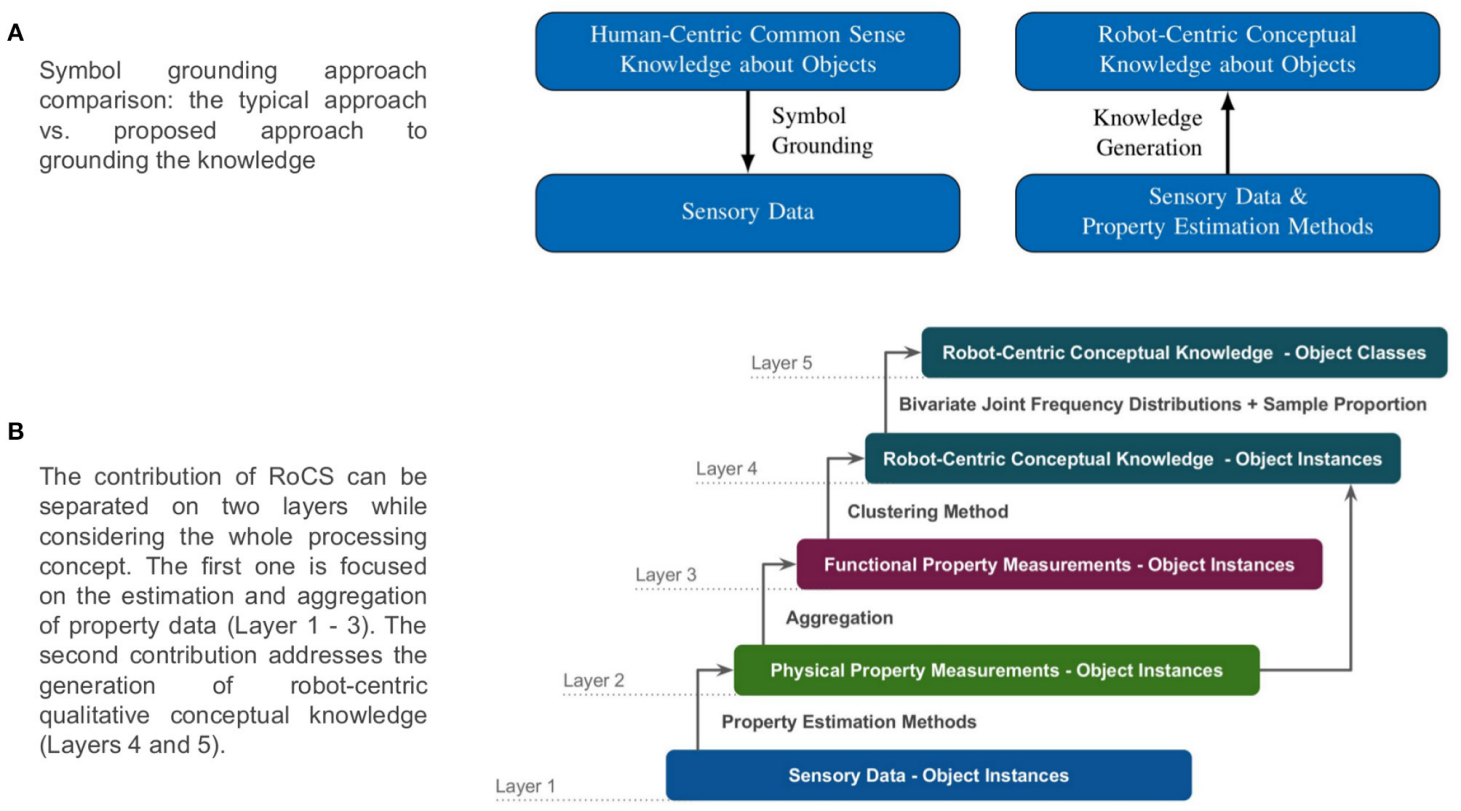
**(A)** represents our symbol grounding approach while figure **(B)** illustrates the process layers for our bottom-up robot-centric knowledge generation.

We propose that such robot-centric conceptual knowledge should be generated in a bottom-up fashion: First, we capture the sensory data about various properties of objects. The sensory data is then processed to estimate *quantitative* measurements of properties observed in objects which are then used to generate property specific *qualitative* measurements. A conceptual knowledge about objects is then generated for given objects on the basis of the qualitative measurements of various properties (see Figure 2B).

#### 1.1.3. Knowledge Representation

For representing conceptual knowledge about objects for substitute selection, we need a formalism that represents the four aspects of conceptual knowledge as stated above: *generalized, relative, subjective*, and *qualitative*. Let us see them one by one. A representation of generalized knowledge should express knowledge about an object category in terms its properties. The generalized knowledge about the object category should be relative to a robot's experience with different instances of the respective category and other categories too. The relativeness of the knowledge also entails that the conceptual knowledge about objects should be updated as the robot acquires experiences with new instances of the known object category or a new object category. As a result a formalism for representing relative knowledge should manifest such experiences.

In order to capture such subjectivity, it is necessary that the knowledge is grounded in the robot's own sensory perception of the properties of objects. A symbol grounding process bridges the gap between symbolic knowledge and sensory perception by creating a correspondence between them. This correspondence either refers to a physical entity in the real-world a.k.a. perceptual anchoring (Coradeschi and Saffiotti, 2003) or assigns a meaning to a symbol by means of a respective sensory-motor process (Harnad, 1990) (*what I sense is what I know*) (see Figure 2A). We noted in section 1.1.1 that we require a formalism to represent qualitative knowledge about objects' properties which can be used by a robot in substitute selection. A similar observation has been made in Rand (1990) which states that when representing an object as a concept, humans usually omit quantitative measurements, but assign what we have termed as *qualitative measurements* to the properties of the object to reflect to what degree that property is present in that object category relative to one's own experience. For instance, a cup is generally *light* weight, *medium* rigid, and can *fully* contain solid or liquid.

### 1.2. Related Work

Since the demand for conceptual knowledge has been increasing in robotic applications, the development of knowledge bases has been undertaken by many researchers around the world (cf. Thosar et al., 2018b). While there exists a multitude of knowledge bases, the question is how many existing knowledge bases about objects conform to the above mentioned requirements: conceptual knowledge base containing knowledge about the objects' properties that is *general, relative, subjective (robot-centric)*, and *qualitative*. In Thosar et al. (2018b), we reviewed existing knowledge bases primarily containing knowledge about household objects and their underlying acquisition system developed for service robotics to address this question. For the review article, we selected 20 papers covering 9 knowledge bases about household objects on the basis of the contents of the paper with respect to the above mentioned requirements and overall impact of the paper on the basis of the number of citations (refer Table 1). Our review resulted in the following conclusions with respect to each building block discussed in the previous section:

**Table 1:** List of selected knowledge bases and their names originally appeared in Thosar et al. (2018b).

**Knowledge base**	**Acronym**
Knowledge processing system for Robots	KNOWROB (Tenorth and Beetz, 2009)
Knowledge Base using Markov Logic Network	MLN-KB (Zhu et al., 2014)
Non-Monotonic Knowledge-Base	NMKB (Pineda et al., 2017)
Open Mind Indoor Common Sense	OMICS(Gupta and Kochenderfer, 2004)
Ontology-based Multi-layered Robot Knowledge Framework	OMRKF (Suh et al., 2007)
OpenRobots Ontology	ORO (Lemaignan et al., 2010)
Ontology-based Unified Robot Knowledge	OUR-K (Lim et al., 2011)
Physically Embedded Intelligent Systems	PEIS (Daoutis et al., 2009)
Knowledge Engine for Robots	RoboBrain (Saxena et al., 2014)

**Conceptual Knowledge:** As our desired conceptual knowledge about an object consists of qualitative knowledge about its physical and functional properties, we reviewed the existing knowledge bases to examine whether such conceptual knowledge was considered. We noted that the majority of the knowledge bases relied on the external human-centric commonsense (universal) knowledge bases such as ConceptNet (Liu and Singh, 2004), WordNet (Fellbaum, 1998) (KnowRob, MLN-KB, OMICS, RoboBrain), Cyc (Lenat, 1995) (PEIS-KB), OpenCyc (Lenat, 1995) (KnowRob, ORO, RoboBrain) and the rest either relied on the hand-coded knowledge (OMRKF, OUR-K) or on knowledge acquired by human-robot interaction (NMKB), for the symbolic conceptual knowledge about objects. Our review concluded that while the existing knowledge bases do contain *general* knowledge about objects, they do not contain qualitative knowledge about their properties as discussed in section 1.1.1. For instance, a *cup* is described in WordNet as *a small open container usually used for drinking; usually has a handle*. The description does not contain qualitative knowledge about various properties such as size, shape, weight, roughness, or rigidity observed in a cup. Moreover, it is worth noting that the knowledge about objects in the existing knowledge bases is universal in nature and thus lacks subjectivity and relativity aspects of knowledge. While having common sense, universal knowledge has its merits, in section 4.4.1, we have discussed an experiment which illustrates the inadequacy of using WordNet in substitute selection without pre-selecting knowledge.

**Knowledge Representation:** Logic based representation formalisms were overwhelmingly used by a majority of the knowledge bases to represent knowledge: OWL-RDF (KnowRob, OMRKF, ORO, OUR-K), Markov Logic Network (MLN-KB), Prolog - Horn Clause (NMKB), Second Order Predicate Logic (PEIS), while database inspired formalisms were used by RoboBrain (Graph Database) and OMICS (Relational Database). Besides representing knowledge about objects, the knowledge bases also focus on representing various uncertainty factors such as noisy sensor information, incomplete knowledge, unknown objects or environment, and inconsistent knowledge. While all the above uncertainty factors are significant, the desired factors such as relativity, and qualitative measures were not formalized while representing knowledge about object properties. For instance, when we think of a cup, although at the abstract level, it is a type of container, the degree of containment is different in a cup for espresso coffee and a cup for tea. Such variation in the containment is not reflected in the representations in the knowledge bases.

**Robot-Centric:** Almost all of the knowledge bases (except for OMICS) addressed the problem of symbol grounding. While the object labels, appearance related properties (shape, size, etc.), and functional properties (KnowRob, MLN-KB, NMKB, PEIS) were grounded in the robot's perception, the reliance on human-centric symbolic knowledge did pose a disadvantage. Since the commonsense knowledge bases are fully human-made, the depth and breadth of the knowledge is not perceivable by a robot due to its limited perception and manipulation capabilities. While a low portion of human-centric knowledge is grounded into robot's limited perception, the majority of the knowledge base remains non-grounded. We believe that such non-grounded knowledge may not be adequate for a robot in a substitute selection task.

It should be noted that the knowledge bases existed independent of the sensory perception. The symbol grounding processes were introduced in the knowledge bases to correspond the sensory perception with relevant symbolic knowledge. In contrast, our proposed approach generates knowledge from the quantitative measurements computed from the sensory data and as a consequence, the knowledge generated from the sensory data for a robot A may differ from the knowledge generated for robot B. This is due to the different sensory capabilities of both the robots, thus reflecting the notion of robot-centricity: object understanding from a first-person perspective.

### 1.3. Contribution

The research work discussed in this article offers a framework for generating robot-centric knowledge about physical and functional properties of objects. Our contribution is 2-fold: we propose

- an approach to extract the sensory data and estimate quantitative measurements related to physical properties of objects;
- an approach to generate robot-centric qualitative conceptual knowledge about objects from the quantitative measurements.

#### 1.3.1. Multi-Modal Physical Property Estimation

Our primary objective is to generate robot-centric conceptual knowledge about objects from object properties based on robot sensory data. In order to realize such a bottom-up approach to generate symbolic knowledge, the first step is to extract the sensory data about an objects' physical and functional properties. In this research work, we primarily focus on extracting sensory data about physical properties from objects. The contribution for physical property extraction from objects is 2-fold:

**Physical properties estimation**: We saw earlier that the methods from the literature on tool selection and substitute selection do estimate one or more physical properties of objects. Besides these two applications, approaches for estimating various physical properties of objects such as rigidity, shape, texture, size, etc. have been proposed for applications such as object recognition/categorization, grasping, and manipulation (Takamuku et al., 2007; Kraft et al., 2009; Sinapov et al., 2009; Spiers et al., 2016; Wu et al., 2016; Kaboli et al., 2017; Kim et al., 2018). In this work, we propose light-weight estimation methods for rigidity, hollowness, size, flatness and roughness, requiring a minimal experimental set-up. Our proposed methods estimate the properties from a single instance at a time and do not require any prior training data for estimation, in contrast to the methods proposed in Wu et al. (2016), Sinapov et al. (2009), Spiers et al. (2016), and Kim et al. (2018).

**Property estimation framework**: In this article, we propose an *extensible* property estimation framework called Robot-Centric Dataset Framework (RoCS) wherein multiple property estimation methods can be used to measure various physical properties and functional properties. Currently, the framework consists of six physical properties and four functional properties where the measurements of functional properties are estimated on the basis of metrics of the physical properties. Our proposed framework is flexible in that it separates the sensory data acquisition from the actual property estimation methods. Such separation allows for redefining the estimation methods with a different set of sensory data than the existing one. Additionally, the proposed framework is also used to create a multi-layered dataset about household objects where the layers denote the different levels of abstraction (Figure 2).

#### 1.3.2. Generation of Robot-Centric Conceptual Knowledge:

**Figure 3:**
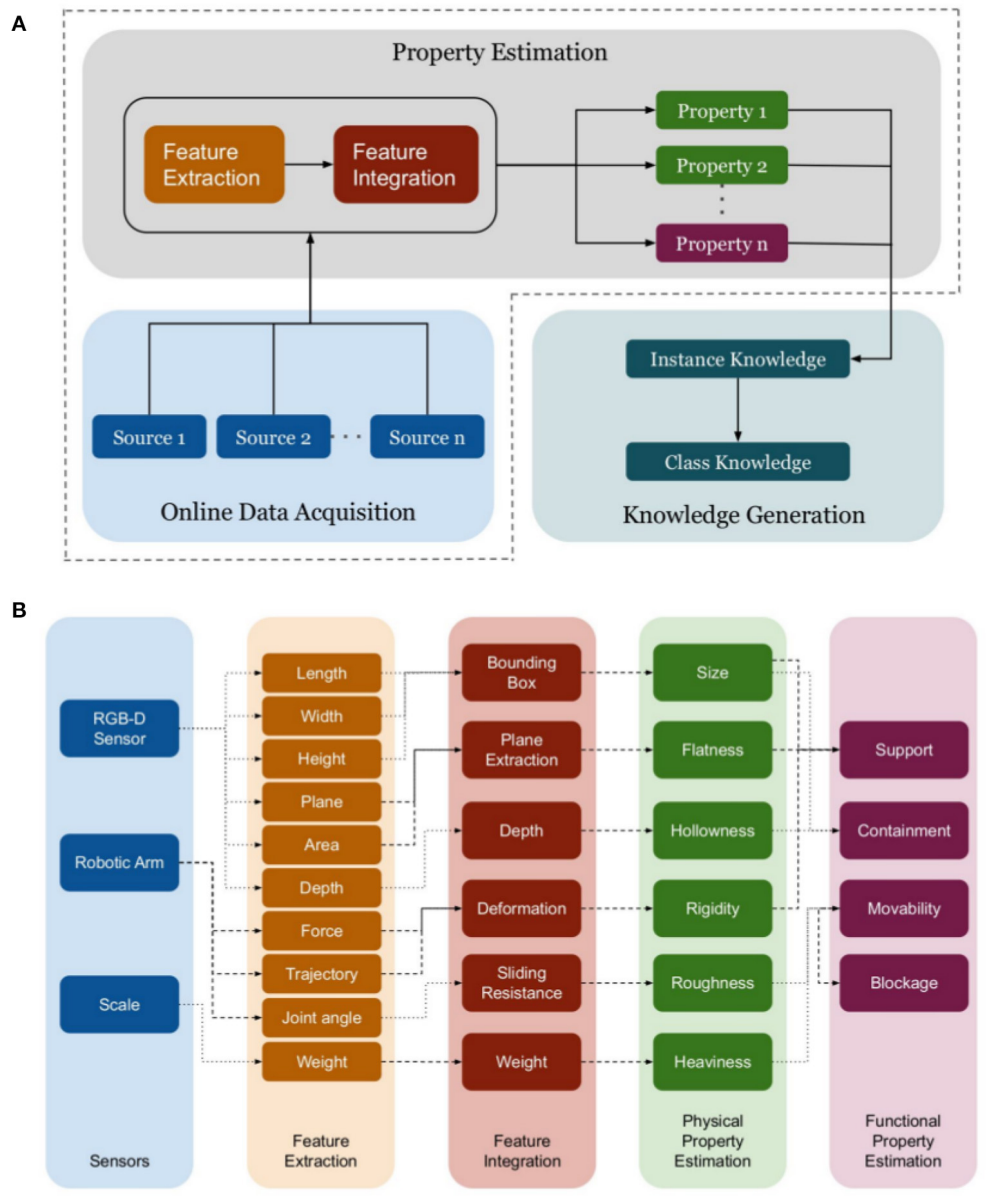
**(A)** RoCS property extraction framework for extracting sensory data related to various properties and generating robot-centric conceptual knowledge about objects; **(B)** Proposed property hierarchy and their dependencies (arrow colors chosen to visually distinguish dependencies).

Our second contribution focuses on the generation of robot-centric conceptual knowledge from the quantitative measurements of object properties. Besides quantitative measurements, a set of symbols representing property labels and object labels are provided a priori for generating knowledge. The proposed knowledge generation method generates knowledge about individual instances which is then used to generate *general* knowledge about object classes as illustrated in Figures 2B, 3 - Layer 4 and Layer 5.

For estimating *qualitative* measurements, a clustering method is used on the quantitative measurements for each property. Each cluster in this case represents a qualitative measurement for the property. As a consequence, the knowledge about an individual instance consists of the respective qualitative measurements of each property. In this manner, each qualitative measure of an instance is grounded into its corresponding quantitative measure of the property while each property symbol is grounded into its estimation method.

In order to generate knowledge about an object class, we propose using the statistical methods Bivariate Joint Frequency Distribution followed by Sample Proportion on the instance knowledge base. We use these statistical methods to model the intra-class variations in an object class. In other words, these methods provide insights into which qualitative measures of a property were observed the most and the least across the various instances of an object class. At the end, an *Attribute-Value Pair* based formalism is used to represent a qualitative measure and its corresponding sample proportion of an object class. The collection of corresponding attribute-value pairs represent knowledge about an object class. We would like to point out here that the proposed bottom-up knowledge generation approach is attributed to the fact that in the absence of any sensory data, the conceptual knowledge base can not be created (Figure 2).

## 2. Property Estimation Framework

The underlying principle behind the proposed framework is to estimate physical and functional properties from a single instance. We have proposed expert-based models for estimating five physical properties namely rigidity, roughness, hollowness, flatness, and size, requiring minimal experimental set-up. In contrast, data-driven models would typically need many training examples for each property which may not be feasible as more properties are added in the framework. Our ultimate vision for the framework is to develop an online platform where researchers from around the world can plug-in estimation methods for the same or new properties, physical and functional, including the documentation on the required experimental set-up. The idea is to allow users to select the estimation methods based on the available hardware at their end in order to acquire robot-centric measurements of object properties.

The Figure 3A illustrates the modular structure of the estimation framework. It primarily consists of two modules: online data acquisition and property estimation. The online data acquisition module is responsible for extracting sensory data about objects. In Figure 3B, the property estimation modules consists of three primary phases as depicted in the figure. In the *feature extraction* phase, the desired features are extracted from the sensory data (see Figure 3B–feature extraction). The extracted features are integrated in the *feature integration* phase to form the primary parameters required for estimating the measurements of the properties (see Figure 3B–feature integration). The last phase consists of computing the quantitative measurements using the proposed expert-based models (see Figure 3B - physical and functional property estimation). The quantitative measurements of the properties are then forwarded to the knowledge generation module for generating conceptual knowledge about objects.

In our framework, the decoupling of data acquisition, feature extraction and feature integration allows the flexibility for redefining the existing property estimation models or proposing estimation models for new properties. For instance, in the current system, hollowness is defined on the basis of depth. However, it can be redefined on the basis of size and depth as well. Such flexibility, in our opinion, is necessary for robot-centric measurement acquisition since sensory and manipulation capabilities vary from robot to robot. As a result, based on the available sensor and manipulation capabilities, a user may re-purpose the features for redefining the properties or design estimation methods for new properties. The desire for flexibility is driven by one of the pressing issues which is interpreting the meaning of the properties. The meaning can be complex where various facets of a property and their relationship to the various parts of an object are perceived and interpreted accordingly or it can be primitive or simplistic. In either case, *the meaning or definition of a property forms a basis for designing a hardware set-up and a subsequent estimation method*.

In the following section, we will discuss in detail our proposed property estimation methods to acquire the quantitative measurements of objects properties. An approach to the generation of robot-centric conceptual knowledge from the acquired quantitative measurements is discussed in section 3. Section 4 discusses the extraction of the dataset from 110 household objects using the proposed framework and the estimation methods (section 4.1) followed by an evaluation of the quality of the acquired dataset (section 4.2), the semantics of property measurements (section 4.3), as well as knowledge base generation and its application in a tool substitution scenario (section 4.4).

### 2.1. Property Estimation

In this section, we will discuss the selection of properties, their definitions, and proposed estimation methods for acquiring their quantitative measurements for a robotic platform. Currently the framework supports six physical properties namely rigidity, roughness, flatness, size, hollowness and weight, and four functional properties namely containment, blockage, movability, and support. In this work, when interpreting the properties, simplistic definitions of the properties were formed which allowed for a minimal set-up and light-weight estimation methods. The intend behind a simplistic approach is that it allows the use of a simple mobile manipulator whose limited capabilities can be exploited. Additionally, such a minimal experimental-set up can easily be reproduced as they do not require high-end robotic platforms. The notion of the physical properties is based on the physical properties in solid-state physics, where they are considered as properties which can be observed, measured, and quantified. We have extended the notion of functional properties in the similar fashion where they are measured and quantified. The selection of these properties are inspired by literature on the tool use in humans and animals (Vauclair and Anderson, 1994; Baber, 2003; Susi and Ziemke, 2005; Hernik and Csibra, 2009; Vaesen, 2012; Biro et al., 2013; Sanz et al., 2013).

In the following, each property is described in a 2-fold manner. First, for each property a general *definition* is provided where we aim for a *simplistic* and *intuitive* characterization for each property. The property definitions considered in this work are not unique. The proposed framework can be extended by plugging in separate estimation methods for the same property based on more complex and/or different characterizations. Second, for each property an *estimation method* is proposed. Note that, although the property definitions are formulated from a human perspective, our ultimate aim is toward enabling a robot to assemble its own understanding about objects, given its own perceptual capabilities in form of vision and manipulation feedback.

**Figure 4:**
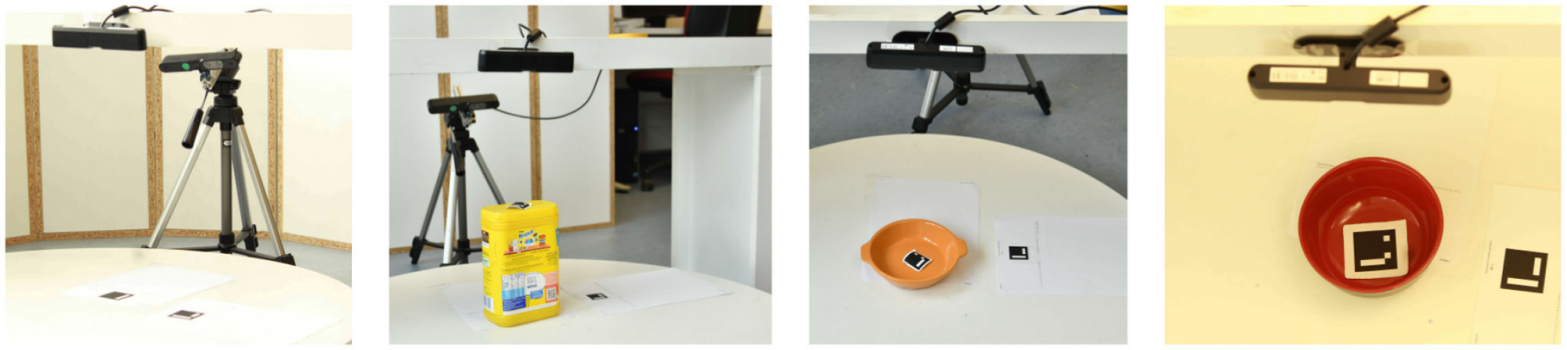
Light-weight experimental setup consisting of two cameras and fiducial markers (Garrido-Jurado et al., 2014), for acquiring physical properties.

**Figure 5:**
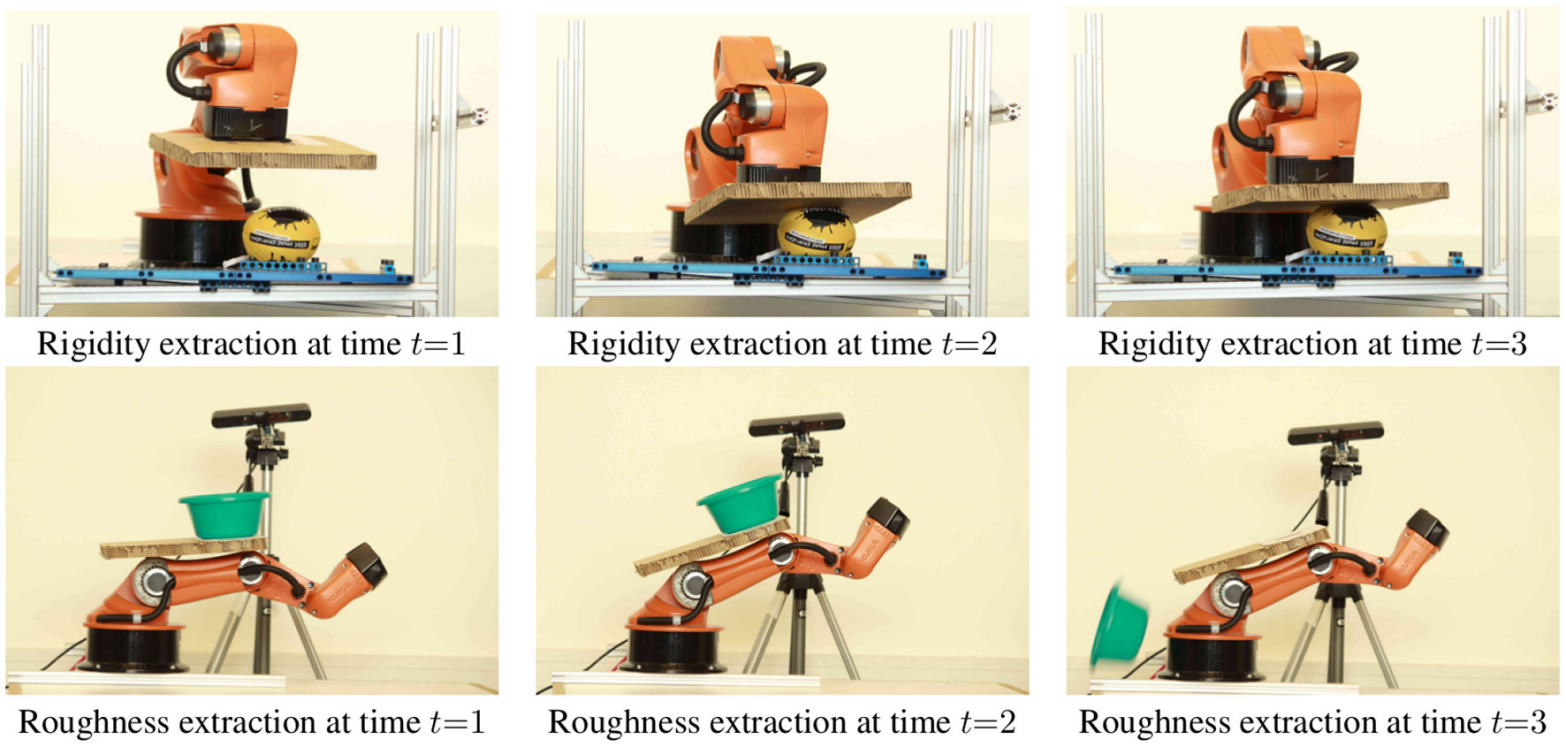
Light-weight experimental setup consisting of a camera-manipulator combination, for acquiring physical property *rigidity* (top row) and *roughness* (bottom row).

Hence, we have derived estimation methods allowing a robot to interpret its *sensory feedback* (Figure 3B) of objects for generating numeric representations of *physical* and *functional* properties. While the presented methods consider features acquired from our robotic platform (Kuka youBot; Bischoff et al. (2011), see Figure 5) and an RGB-D sensor (Asus Xtion Pro; Swoboda, 2014), we aim to propose a light-weight setup (Figure 4) and methods that are transferable and adoptable to other robotic platforms by considering common hardware interfaces and data representations such as images, point clouds, or joint states of robotic manipulators.

We may summarize, that the following proposed estimation methods represent a possible mechanism to express these properties to achieve a continuous-valued property feedback. Depending on the robot capabilities, various estimation methods can be introduced based on different modalities such as vision, tactile, or auditory feedback. Therefore, first and foremost, the following methods serve as a possible basis to receive feedback of the targeted properties from a robotic perspective.

### 2.2. Physical Properties

In this work, we have selected *flatness, hollowness, size, roughness, rigidity*, and *heaviness* as physical properties given their significance reported in the literature on tool use in humans and animals (Vauclair and Anderson, 1994; Baber, 2003; Biro et al., 2013; Emery, 2013). The main inspiration behind selecting these properties was the prominent roles these properties played in various tool use scenarios in humans and animals alike, as widely reported in the literature. For instance, human infants begin exploring their abilities to use any object by studying and interacting with it to understand its *weight, texture*, and *shape* (Vauclair and Anderson, 1994). While designing and manufacturing a tool, humans and animals alike pay closer attention to the properties such as *shape, size, rigidity, roughness*, and *heaviness* (Baber, 2003, Chapter 6). It has been observed that wild animals select the tools based on the *size, shape* or mechanical properties such as *strength, hardness* (Biro et al., 2013). For example, otters have been observed carrying *flat* rock on their chest which they use to break the shellfish (Emery, 2013). On the other hand, researchers found that the monkeys are able to select the hardness of the stone with respect to the hardness of the nut they want to cut open (Boesch, 2013).

In the following, we provide a *definition* for each physical property and subsequently an *estimation method* is proposed for each property. Note that, across all estimation methods, we assume that an object is placed in its most natural position, for instance, a cup is most commonly placed in such a way that its opening points upwards. Furthermore, we aim at a bounded property value, i.e., an estimated property value that is mapped into a [0, 1] interval in order to enable a subsequent unbiased property analysis which is not affected by object-specific characteristics or scales. Note that, as a prerequisite, each object is segmented a priori through a table-top object segmentation procedure, particularly for the *size, flatness*, and *hollowness* property. As a result, estimated property values of each object are captured through the given capabilities of the robot in form of vision-based (e.g., featuring particular image, point cloud resolution, or viewpoint) as well as manipulation-based (e.g., featuring particular joint-states, limits, or force-feedback) input. Consequently, these property values are originated from a robot-centric perspective on the perceived objects.

#### 2.2.1. Size Property

**Definition:** *Size* of an object is defined intuitively by the object's spatial dimensionality in form of *length, width*, and *height*.

**Estimation:** The size of an object is defined by the length, width, and height. As it therefore can be estimated by determining an object's bounding box, we use an RGB-D sensor to obtain point clouds of the object from a lateral perspective. Using marker detection to define a region of interest (ROI), we segment the object and transform its point cloud to an axis-normal representation, i.e., the z-axis is aligned with the object's height. Subsequently, an axis-aligned bounding box is approximated given the extracted object point cloud. The size = [length, width, height] of an object is directly derived from the object point cloud as distances between the minimal and maximal value in each spatial dimension of the bounding box. In order to retrieve a bounded property value range [0, 1] for the property *size* (*si*), each spatial dimension of size [length, width, height] is normalized by the largest dimension of the object (see Equation 1). As a result, *si* is defined as a three dimensional property.

(1) $$\begin{array}{l} si=\left[l=\frac{\text{length}}{\max\left(\text{size}\right)},w=\frac{\text{width}}{\max\left(\text{size}\right)},h=\frac{\text{height}}{\max\left(\text{size}\right)}\right] \end{array}$$

Note that, *max*(*size*) merely abbreviates *max*(*length, width, height*).

#### 2.2.2. Flatness Property

**Definition:** As *flatness* describes a particular aspect of an object's shape, we define it as the ratio between the area of an object's greatest horizontal plane and its overall surface area. For instance, a sheet of paper features an upper bound of *flatness* whereas a ball features a lower bound of *flatness*.

**Estimation:** The *flatness* value of an object is estimated similarly to its *size*: We firstly observe the object from above (Figure 4) and extract its greatest plane using RANSAC [RAndom SAmple Consensus; Fischler and Bolles, 1981]. In order to increase the confidence, a candidate plane is only selected if at least 95% of the surface normal vectors of the plane points are directed in the same direction, up to a threshold. In this manner, round surfaces (as they may be observed in *balls*) are rejected and subsequently a *flatness* value of zero is assigned to the considered object. Furthermore, if the candidate plane *p* is accepted, the plane size |*p*|, i.e., the number of object points corresponding to *p*, is divided by the total number of points |*o*| representing the observed object *o* in order to obtain a bounded numeric measure of its *flatness* *fl* (Equation 2). Consequently, the retrieved *flatness* property is bounded within a value range of [0, 1].

(2) $$\begin{array}{l} fl=\frac{\left|p\right|}{\left|o\right|} \end{array}$$

#### 2.2.3. Hollowness Property

**Definition:** *Hollowness* is the amount of visible cavity or empty space within an object's enclosed volume. It contrasts *flatness* as it focuses on a further particular aspect of an object's shape.

**Estimation:** *Hollowness* contributes to the characterization of object shape. According to its definition, an object may enclose a volume which is not filled. For the sake of simplicity, we measure the internal depth *d*, which resembles the enclosed volume, and height *h* of an object *o*: the ratio defines the *hollowness* value. In order to retrieve a reasonable measure of object's depth and height, a two camera and fiducial marker (Garrido-Jurado et al., 2014) setup is introduced as illustrated in Figure 4. Given the side camera view, the height *h* of an object can be obtained by estimating the respective bounding box (see section 2.2.1). In order to retrieve depth, two fiducial markers {*m*_*r*_, *m*_*h*_} are introduced (see samples in Figure 4): *m*_*r*_ serves as global reference and is placed next to the object; *m*_*h*_ is placed inside the hollow volume of the object. Exploiting the top camera height *c*_*t*_ perpendicularly pointed to the object, the distances *d*_*r*_ = ||*m*_*r*_ − *c*_*t*_|| and *d*_*h*_ = ||*m*_*h*_ − *c*_*t*_|| can be obtained. Given object height *h* and the distances *d*_*r*_ and *d*_*h*_, hollowness *ho* can be approximated as shown in Equation (3b), where *b* (Equation 3a) is introduced to consider the base height of the object, i.e., distance between the table (global reference plane) and the bottom inside the object's hollow volume.

(3a) $$\begin{array}{ll} b & =d_{r}-d_{h} \end{array}$$

(3b) $$\begin{array}{ll} ho & =\frac{h-b}{h} \end{array}$$

Note that, *ho* is inherently bounded within the interval [0, 1]. Furthermore, the proposed method may be susceptible to noise originated in the point clouds from which the bounding box was approximated to infer the object's height *h*. Hence, if the difference between an object's height *h* and distance *d*_*h*_ (fiducial marker inside the object) is smaller than 1cm it is cumbersome to differentiate between sensor noise and the actual *hollowness* due to the low signal-to-noise ratio. To sanitize the property in such situations (particularly in case of flat objects), default value of zero is assigned.

#### 2.2.4. Heaviness Property

**Definition:** Following our basic premise of using straight forward definitions, we borrow the definition of *heaviness* from physics: the object's *heaviness* is the force acting on its mass within a gravitational field.

**Estimation:** *Heaviness* *he* of an object *o* can be directly derived by weighing an object with a *scale* (Equation 4); a scale with a resolution of 1g provides an adequate precision for our scenario. Note that, *he* is normalized by the carrying capabilities of the robotic arm.

(4) $$\begin{array}{l} he=scale\left(o\right) \end{array}$$

While it may require additional hardware, a robot may lift an object and calculate the *heaviness* by converting the efforts observed during the process in each of its joints.

#### 2.2.5. Rigidity Property

**Definition:** *Rigidity* of an object is defined as the degree of deformation caused by an external force vertically operating on it.

**Estimation:** *Rigidity* of an object is estimated using a robotic arm. The arm is equipped with a planar end-effector that is used to vertically exert a force onto an object until predefined efforts in the arm's joints are exceeded, see Figure 5; by setting the predefined efforts to the limits of the robotic arm, the final rigidity value is specific to the robot executing the estimation method. During this process we record the trajectory *tr*(*t*) of the arm as well as the efforts in all of its joints. By analyzing them using an adaptive threshold-checking, we detect the first contact of the end-effector with the object *o* at time *t*_0_. Using the final position of the arm when the efforts are exceeded at *t*_1_, we can calculate the deformation *def* of an object as the vertical movement of the end-effector, that is, its movement along the *z*-axis between *t*_0_ and *t*_1_:

(5a) $$\begin{array}{ll} def\left(o\right) & =tr_{z}\left(t_{0}\right)-tr_{z}\left(t_{1}\right) \end{array}$$

(5b) $$\begin{array}{ll} ri & =\frac{def\left(o\right)}{h} \end{array}$$

In that way, the deformation *def*(*o*) is nothing but the distance the arm pushed into the considered object. For rigid objects, this deformation is zero while it is increased continuously for non-rigid objects. Finally, we normalize the deformation by the height *h* of the object to obtain its *rigidity* value *ri*. As we use a distance as a measure of an object's deformation, *def*(*o*) will always be positive. Furthermore, as an object may not be deformed more than its own height, the value of *ri* is naturally bound to the interval of [0, 1].

#### 2.2.6. Roughness Property

**Definition:** *Roughness* provides information about an object's surface. Therefore, we simplify the physical idea of friction and define *roughness* as an object's resistance to sliding.

**Estimation:** *Roughness* *ro* requires interaction as well to measure an object's resistance to sliding. The robotic arm is exploited to act as a ramp on which the considered object is placed, see Figure 5. Starting horizontally, with an initial angle of *a*_*i*_ = 0°, the ramp's angle is increased and thereby causes an increasing gravitational force pulling the object down the ramp. When the object begins sliding, a fiducial marker that is a priori placed underneath the object, is unveiled and subsequently detected. As this means that the object's sliding resistance is exceeded, the ramps' angle *a*_*r*_ is observed and exploited as a measure of *roughness* as shown in Equation (6). In this setup, a 90° ($\frac{\pi }{2}$) ramp angle represents the upper bound that induces an object to slide. Hence, it is used to normalize *roughness* value *ro* within [0, 1].

(6) $$\begin{array}{l} ro=\frac{\left|a_{i}-a_{r}\right|}{\frac{\pi }{2}} \end{array}$$

### 2.3. Functional Properties

In contrast to physical properties, functional properties describe the functional capabilities or affordances (Gibson, 1986) of objects. It is proposed that functional properties do not exist in isolation, rather certain physical properties are required to enable them (Baber, 2003, Chapter 5). In tool use, functional properties play an important role especially when perceiving an object as a possible tool since humans in general characterize an object in terms of its functional properties rather than its physicality (Gibson, 1986; Hartson, 2003). The question is how does a functional property or affordance emerge? In other terms, what are the required qualifications for an ability to be recognized as a functional property or an affordance? Various theories have been proposed to address this question (Gibson, 1986; Hartson, 2003; Osiurak et al., 2017) and among them is a theory proposed by Kuhn (2007). Kuhn (2007) suggests that *image schema* (such as *LINK, CONTAINER, SUPPORT*, and *PATH*) capture the necessary abstractions to model affordances. *Image schema* is a theory proposed in psychology and cognitive linguistics and it concerns with a recurring pattern abstracted from the perceptual and motor processes. Some of the examples of image schema are *containment, support, path*, and *blockage*. These form the basis for functional abilities to *contain, support, move*, and *block* (Figure 3B).

It is suggested by Baber (2003) that a certain assemblage of physical properties are essential prerequisites to enable a functional property and such knowledge is used by humans and animals alike in tool selection. We have exploited this notion and have designed our substitute selection approach (Thosar et al., 2020) around it. Therefore, our proposed knowledge generation approach follows the same suit, where each functional property is defined in terms of its associated enabling physical properties. In the following, we provide the definitions of the functional properties and their corresponding estimation methodology.

#### 2.3.1. Support Property

**Definition:** *Support* describes an object's capability to support, i.e., to carry another object. Therefore, an object is attributed with *support*, if other objects can be stably placed on top of the supporting object. Consequently, the physical properties *size, flatness*, and *rigidity* are enabler of *support*.

**Estimation:** *Support* requires to consider three aspects of an object. Firstly, the considered object needs to be rigid. Secondly, for carrying another object, the sizes of both may feature similar spatial proportions. Thirdly, the object's shape needs to be sufficiently flat in order to enable the placing of another object on top of it. Consequently, *size, flatness*, and *rigidity* are considered as core elements of the *support* property, Equation (7).

(7) $$\begin{array}{l} su=\left[si,fl,ri\right] \end{array}$$

#### 2.3.2. Containment Property

**Definition:** An object is attributed with *containment* if it is capable to enclose another object to a certain degree. This property is enabled by *size* and *hollowness*.

**Estimation:** *Containment* property requires to consider two aspects. In order to contain something, an object needs to be hollow. On the other hand, it's *size* itself needs to be respected when considering whether it can contain another object. Thus, the value of the object's *containment* *co* property is formed by combining its *size* and *hollowness* property values, Equation (8).

(8) $$\begin{array}{l} co=\left[si,ho\right] \end{array}$$

#### 2.3.3. Movability Property

**Definition:** *Movability* describes the required effort to move an object. The physical properties *roughness* and *heaviness* affect the *movability* of an object. As a result, we may interpret that *movability* is affected by these physical properties.

**Estimation:** *Movability* is based on a robot's primary ways of moving objects: either by lifting or pushing. In both cases, *heaviness* of an object is affects the *movability* of an object. Additionally, when pushing an object, its sliding resistance expressed in form of *roughness* (see Figure 5), needs to be considered as well. Therefore *movability* property *mo* constitutes of *heaviness* and *roughness*, Equation (9).

(9) $$\begin{array}{l} mo=\left[he,ro\right] \end{array}$$

#### 2.3.4. Blockage Property

**Definition:** *Blockage* describes the capability of an object of being impenetrable, i.e., the object cannot be moved by other objects, therefore it stops the movement of other encountered objects. Note that, given the set of physical properties, we can interpret that the *blockage* property is related to *roughness* and *heaviness* of an object as these properties affect the intensity of being capable to block another object. According to the property hierarchy (Figure 3B), *blockage* is directly related to its counterpart, i.e., the *movability* property.

**Estimation:** *Blockage* of an object can be derived from its *movability*. According to its definition, *blockage* property *bl* states to which degree an object is able to stop another object's movement. Thus, the object itself needs to be not movable by the other object, which is the inverse of its *movability*, Equation (10).

(10) $$\begin{array}{l} bl=-mo=\left[-he,-ro\right] \end{array}$$

## 3. Generation of Robot-centric Conceptual Knowledge

We propose an unsupervised approach to generate symbols required to represent conceptual knowledge about objects from the perception data estimated using the framework described in the previous section. In this section, we discuss the proposed bottom-up knowledge generation process to obtain robot-centric knowledge about object instances and object classes (see., Figure 2B, layer 4 and 5).

For generating robot-centric conceptual knowledge, the data about the objects' physical and functional properties is processed in two stages: *sub-categorization* and *conceptualization*. In the sub-categorization process, the non-symbolic continuous data of each property is transformed into symbolic data using a clustering algorithm such as *k*-means. The cluster representation of the numerical values of the property data can also be seen as a symbolic qualitative measure representing each cluster. Consequently, the number of clusters describes the granularity with which each property can qualitatively be represented. In case of a high number of clusters, an object is described in finer detail. In contrast, a lower number of clusters suggest a coarse description of an object. For instance, the numerical data about the *rigidity* of the object instances of *ceramic cup*, when clustered into three clusters, can be represented as *rigidity* = {*soft, medium, rigid*}. Note, however, that through the clustering process, the symbols are usually not ordinal but rather categorical. At the end of the *sub-categorization* process, each object is represented in terms of the qualitative measures for each property. The conceptualization process gathers the knowledge about all the instances of an object class and represents the knowledge about an object class. Initially, the knowledge about objects is represented using *bivariate joint frequency distribution* of the qualitative measures of the properties in the object instances. Next, conceptual knowledge about objects is calculated as a sample proportion of the frequency of the properties across the instances of a class. In the following, we have provided the formal description of the knowledge generation process described above.

Consider **O** as a given set of object classes where (by abuse of notation) each object class is identified with its label. Let each object class *O* ∈ **O** be a given set of its instances. Let ⋃**O** be a union of all object classes. Let **P** and **F** be the given sets of physical properties' labels and a set of functional properties' labels respectively. By abuse of notation, each physical and functional property is identified with its label. For each physical property *P* ∈ **P** as well as for a functional property *F* ∈ **F**, sensory data is acquired from each object instance *o* ∈ ⋃**O**. Let Υ_*P*_ and Υ_*F*_ represent functions which maps each object instance to its measured sensory value of a physical property *P* and a functional property *F* respectively. Let *P*_*n*_ and *F*_*n*_ represent sets such that *P*_*n*_ and *F*_*n*_ are the images of Υ_*P*_ and Υ_*F*_, respectively.

### 3.1. Sub-categorization – From Continuous to Discrete

The sub-categorization process is performed to form (more intuitive) qualitative measures to represent the degree with which a property is reflected by an object instance. It is the first step in creating symbolic knowledge about object classes where the symbols representing the qualitative measures of a physical or a functional property reflected in an object instance are generated unsupervisedly by a clustering mechanism. A qualitative measure of a physical property is referred to as a physical quality and that of a functional property as a functional quality.

In this process, *P*_*n*_ and *F*_*n*_ representing measurements of a physical property *P* ∈ **P** and a functional property *F* ∈ **F**, respectively extracted from *n* number of object instances is categorized into a given number of discrete clusters η using a clustering algorithm. Let ∇_*P*_ and ∇_*F*_ be partitions of the sets *P*_*n*_ and *F*_*n*_ after performing clustering on them. Let *P*_η_ and *F*_η_ be the sets of labels, expressing physical qualities and functional qualities, generated for a physical property *P* ∈ **P** and a functional property *F* ∈ **F** respectively. Given the label for a property, the quality labels are generated by combining a property label *P* and a cluster label (created by the clustering algorithm). For example, in *size* = {*small, medium, big, bigger*}, *size* is a physical property and *small, medium, big, bigger* are its physical qualities. Note that, these given physical quality labels are only provided for illustration purposes of the property qualitative measures; however, the quality labels for a property *size* are internally represented as {*size*_1, *size*_2, *size*_3, *size*_4}. At the end of the sub-categorization process, the clusters are mapped to the generated symbolic labels for qualitative measures. Note that the number of clusters essentially describes the granularity with which each property can qualitatively be represented. A higher number of clusters suggest that an object is described in a finer detail, which may obstruct the selection of a substitute since it may not be possible to find a substitute which is similar to a missing tool down to the finer details.

### 3.2. Attribution – Object Instance Knowledge

The attribution process generates knowledge about each object instance by aggregating all the physical and functional qualities assigned to the object instance by the *sub-categorization* step. In other terms, the knowledge about an instance consists of the physical as well as functional qualities reflected in the instance. Let **P**_η_ and **F**_η_ be the families (sets) of sets containing the physical quality labels *P*_η_ and the functional quality labels *F*_η_ for each physical property *P* ∈ **P** and functional property *F* ∈ **F**, respectively. Thus, each object instance *o* ∈ ⋃**O** is represented as a set of all the physical as well as functional qualities attributed to it which are expressed by a symbol *holds* as: *holds* ⊂ ⋃**O** × ⋃(**P**_η_ ∪ **F**_η_). For example, knowledge about the instance *plate*_1_ of a *plate* class can be given as, *holds*(*plate*_1_, *medium*), *holds*(*plate*_1_, *harder*), *holds*(*plate*_1_, *can*_*support*) where *medium* is a physical quality of *size* property, *harder* is a physical quality of *rigidity* property and *can_support* is a functional quality of *support* property.

### 3.3. Conceptualization – Knowledge About Objects

**Figure 6:**
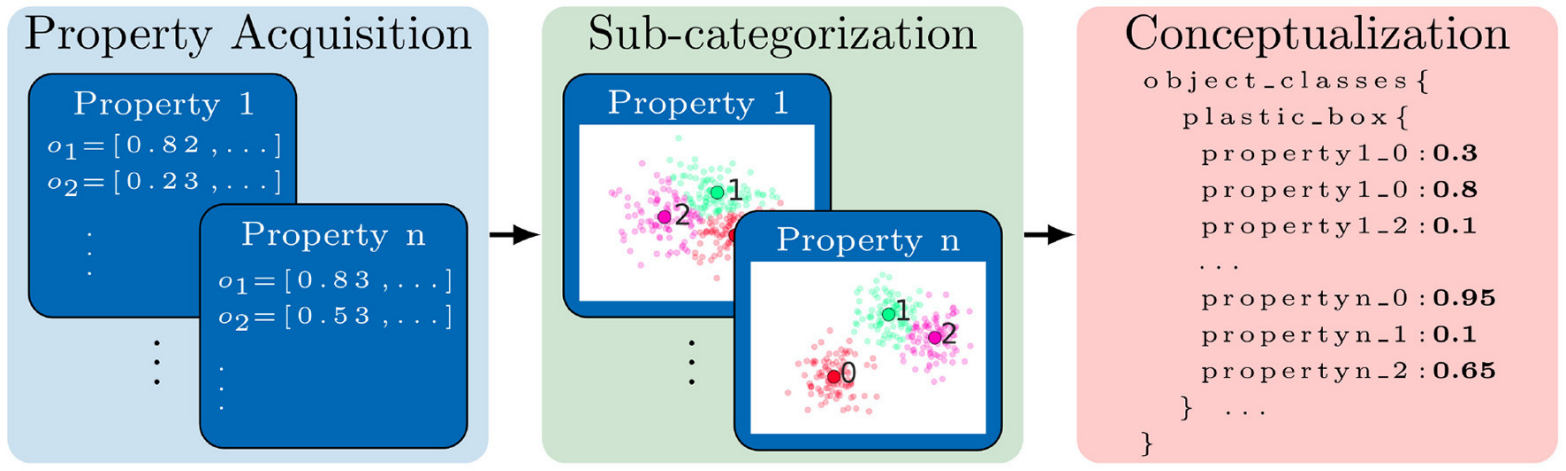
The robot-centric conceptual knowledge generation process is illustrated where acquired continuous property data of objects {*o*_1_, *o*_2_...} is sub-categorized into multiple clusters. Using Bi-variate joint frequency distribution and sample proportions conceptual knowledge about object classes (e.g., *plasti_box*) is generated.

The conceptualization process aggregates the knowledge about all the instances of an object class. The aggregated knowledge is regarded as conceptual knowledge about an object class. Let **O**_*KB*_ be a knowledge base about object classes where each object class *O* ∈ **O**. Given the knowledge about all the instances of an object class *O*, in the conceptualization process, the knowledge about the object class *O*_*K*_ ∈ **O**_*KB*_ is expressed as a set of tuples consisting of a physical or a functional quality and its proportion (membership) value in the object class. A tuple is expressed as 〈*O, t, m*〉 where *t* ∈ ⋃**P**_η_ or *t* ∈ ⋃**F**_η_ and a proportion value *m* is calculated using the following membership function expressed by a conditional probability: *m* = *P*(*holds*(*o, t*)|*o* ∈ *O*). The proportion value allows to model the intra-class variations in the objects. For example, knowledge about object class *table* {〈*plate, harder, 0.6*〉, 〈*plate, light_weight, 0.75*〉, 〈*plate, less_hollow, 0.67*〉, 〈*plate, hollow, 0.33*〉, 〈*plate, more_support, 0.71*〉}, where the numbers indicate that, for instance, physical quality *harder* was observed in 60% instances of object class *plate*. At the end of the conceptualization process, conceptual knowledge about an object class is created which is represented in a symbolic fuzzy form and grounded into the human-generated or machine-generated data about the properties of objects. The knowledge about objects is then used to determine a substitute from the existing objects in the environment. The Figure 6 illustrates graphically the main processes of *Sub-categorization* and *Conceptualization*.

## 4. Experimental Evaluation

In the following evaluation, multiple experiments are conducted to evaluate the proposed approach on different semantic levels: From the property estimation of real world objects to an eventual application scenario in the context of tool substitution.

For this purpose, we introduce the RoCS dataset containing estimated physical and functional properties of objects in section 4.1. With the dataset, we conduct an evaluation on the physical object properties investigating the stability of the estimation methods, the coverage w.r.t. the range of characteristics captured by selected dataset, and the correlation among properties in section 4.2. Using *k*-means clustering (Lloyd, 1982) on functional object properties in sections 4.3 and 3, we show that the chosen properties may allow to discriminate instances of different object classes and identify the inter-class similarities. Finally, section 4.4 shows the applicability of the dataset by learning a model from the generated conceptual object knowledge given the estimated properties and applying it to a tool substitution scenario under real world conditions.

### 4.1. RoCS Dataset

For the sake of a thorough evaluation of our conceptual framework the Robot-Centric dataSet (RoCS) is introduced. Note that we propose a Robot Operating System (ROS) (Koubâa, 2017) based implementation to acquire object data used in the following evaluation. In the following, we briefly introduce the hardware setup and procedures for acquiring raw object data, describe its parameters (e.g., thresholds) and the contents of the final dataset.

#### 4.1.1. Hardware Setup

Figure 3 illustrates the required sensors as data sources. For visual and non-invasive estimation methods, RGB-D sensors are required. More specifically, the *size* property requires a lateral view on objects while the *hollowness* property relies on a birds-eye view. Hence, we employ two Asus Xtion Pro depth sensors (Swoboda, 2014) (see Figure 4). To estimate the physical properties *rigidity* and *roughness*, a robotic arm is required to interact with objects. In this interaction the proposed property estimation methods require arm joint state values which are generally provided by manipulators, such the one we use, a Kuka youBot (Bischoff et al., 2011) manipulator. Finally, a common kitchen scale with a resolution of 1g is used to estimate the weight and *heaviness* of objects.

#### 4.1.2. Object Property Acquisition Procedure

**Figure 7:**
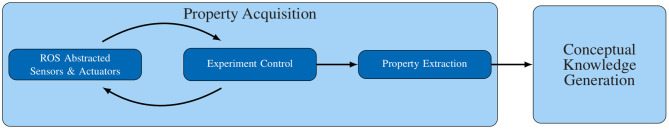
Data flow within the dataset creation framework.

Using the described hardware, we implemented a ROS-based framework to estimate the physical and functional properties of objects. A schematic overview on the framework is given by Figure 7.

The interface for operating sensors and actuators is provided to our framework by ROS. This interface is used by different experiments for observing and interacting with objects to acquire the necessary sensory data. Together, both blocks (*ROS Abstracted Sensors & Actuators* and *Experiment Control*) form a control loop enabling to generate feature data (see Figure 3B). According to the selected properties four control loops are implemented as separate experiments. The first experiment is non-invasive and gathers the visual feature data required for *hollowness, flatness*, and *size*; Figure 4 illustrates the camera setup. Initially a table-top object detection is introduced that uses a RAndom SAmple Consensus (RANSAC) based plane fitting approach in order to detect object candidates on the table. The RANSAC algorithm is parameterized with a leaf size of 0.0025 m, a maximum of 10^4^ iterations and a 0.02 m distance threshold between points and the estimate plane model. Note that, RANSAC is also used in this experiment for segmenting planes for the property *flatness*. Furthermore, fiducial markers (ArUco Library; Garrido-Jurado et al., 2014) with sizes of 14 and 3 cm are used for the *hollowness* property. The second experiment uses the robotic arm to deform objects to facilitate the estimation of *rigidity* (see section 2.2.5). We set the efforts to exceed in each joint to ±8 Nm. Within the third experiment, the robotic arm is used as a ramp to estimate an object's *roughness* (see section 2.2.6). To achieve an appropriate resolution, the angular speed of the joint lifting the ramp is set to 0.05 rad/s. Finally, the last experiment employs a kitchen scale with a resolution of 1g to estimate the objects' weight. Following the *Experiment Control*, the individual estimation methods process the generated feature data as described in section 2.1 to produce physical and functional property values of the considered object. Finally, this data can be accumulated for a set of objects and further processed to generate conceptual knowledge.

#### 4.1.3. Dataset Structure

**Figure 8:**
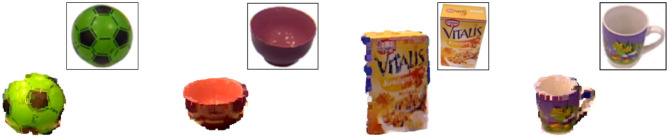
RoCS dataset samples: Point cloud and RGB images of a *ball, bowl, paper box*, and *cup* (for visualization purposes, images are scaled and 3D points are magnified).

For the RoCS dataset we consider 11 different object classes (*ball, book, bowl, cup, metal_box, paper_box, plastic_box, plate, sponge, to_go_cup*, and *tray*) featuring various object characteristics – from appearance to functional purpose. Each class consists of 10 unique object instances that leads to a total number of 110 object instances; Figure 8 illustrates sample object instance of RoCS dataset.

In order to evaluate the performance of the proposed property estimation methods, such as stability, for each object instance we capture 10 repetitions without modifying the setup. As a result we captured 1,100 object observations for which physical and functional property values are generated. The dataset is publicly available at https://gitlab.com/rocs_data/rocs-dataset.

### 4.2. Property Estimation

The objective of the first part of the evaluation is to investigate the property estimation methods as described in section 2.1. At this level, we only focus on physical properties as functional properties are built on the basis of an object's physical properties. First, we analyze the stability of the estimation methods to determine how deterministic and reproducible the data acquisition is for each property and object. Furthermore, we explore the coverage of our data set to determine the variance and range of objects reflected in the different classes and properties. Lastly, we inspect the correlation among different properties in our data.

#### 4.2.1. Estimation Stability

The abstraction process from raw sensor data to symbolic object property knowledge requires a stable processing. However, noise is naturally affecting data when working with sensors and real world objects.

**Figure 9:**
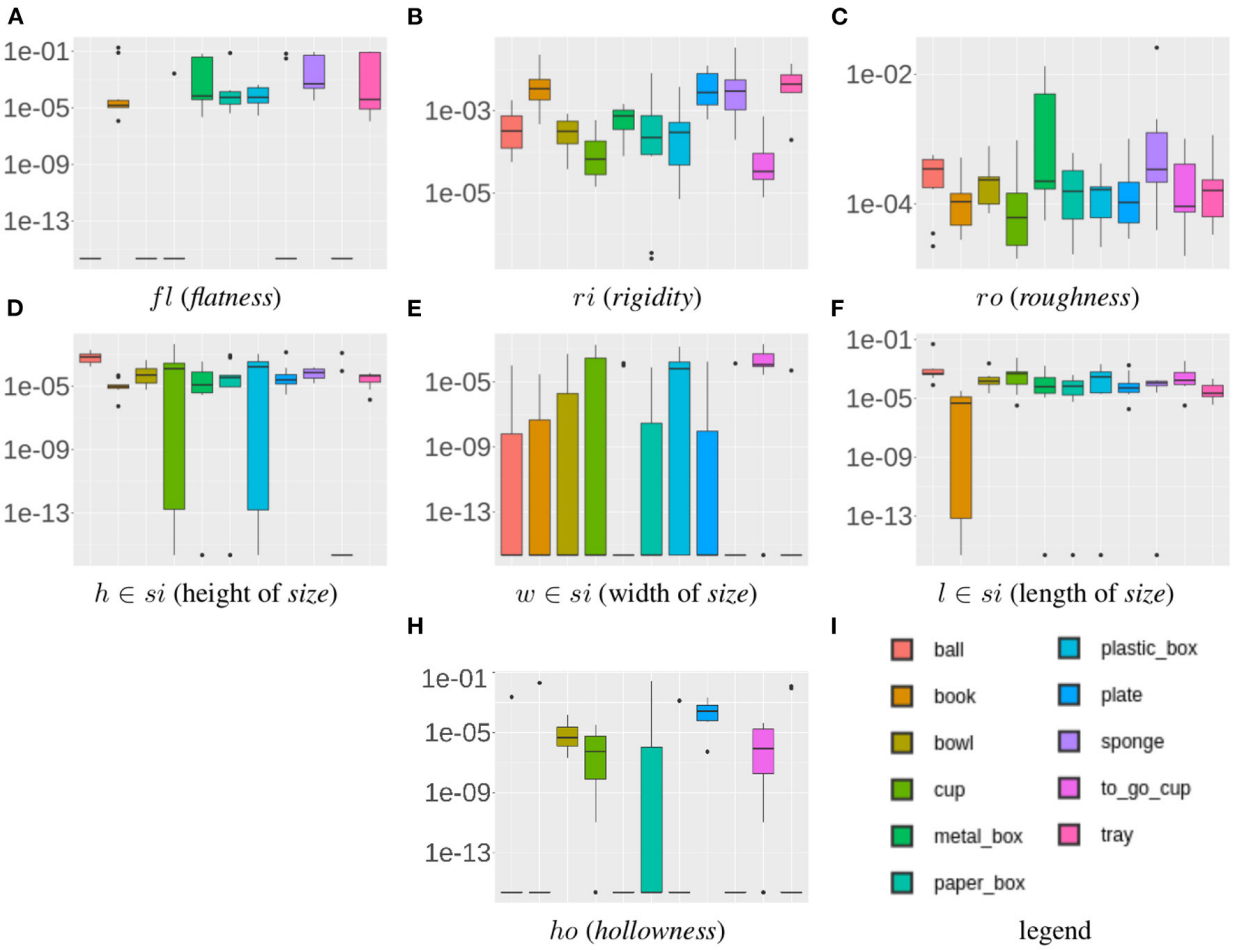
Mean variance for physical properties [*fl, ri, ro, si, he, ho*] illustrated in form of a Box plot (in log-scale to provide insights of respective intra property variances compared to linear-scale shown in Table 2). Note that, in order to be able to display all variances (including zero) in log-scale, we add an epsilon on each value before computing log. Heaviness is excluded as all variance values are zero for this property due to the resolution of the scale.

To compensate for the caused uncertainty, each RoCS object instance consists of 10 repetitions. We use these in the following to analyze the stability of the proposed property estimation methods. For that, the variance of each physical property of each object instance is analyzed. More specifically, given the 10 repetitions of a particular object instance for each of its physical properties, we calculate the variance of the property values of its 10 repetitions. As the measurements of 6 physical properties are based on 8 features, we obtain 8 values per object instance and therefore 880 values in total. We further reduce the data, by calculating the mean of the object variances for a particular object class and property as shown in Table 2, whereas Figure 9 illustrates the variances of all object instances within one object class as box plots; the colored middle box represents 50% of the data points and the median of the class is indicated by the line that divides the box.

**Table 2:** Mean variance for each physical property.

**Class**	**Flatness**	**Rigidity**	**Roughness**	**Size_length**	**Size_width**	**Size_height**	**Heaviness**	**Hollowness**	**Class_mean**
Ball	0	0.00053	0.00032	0.00538	0.00001	0.00083	0	0.00023	0.00091
Book	0.02554	0.00583	0.00015	0.00001	0.00001	0.00002	0	0.002	0.00419
Bowl	0	0.00037	0.00025	0.00038	0.00006	0.00012	0	0.00003	0.00015
Cup	0.00026	0.00015	0.00017	0.00098	0.0003	0.00079	0	0.00001	0.00033
Metal_box	0.01939	0.00074	0.0039	0.00028	0.00002	0.00007	0	0	0.00305
Paper_box	0.00747	0.00115	0.00021	0.00011	0.00002	0.00017	0	0.0035	0.00158
Plastic_box	0.00015	0.00071	0.00016	0.00056	0.00021	0.0003	0	0.00013	0.00028
Plate	0.00971	0.00481	0.00022	0.0003	0.00003	0.00017	0	0.0005	0.00197
Sponge	0.02503	0.00705	0.00313	0.0001	0.00001	0.00008	0	0	0.00443
to_go_cup	0	0.00016	0.00031	0.00061	0.00044	0.00013	0	0.00001	0.00021
Tray	0.03486	0.00569	0.00024	0.00005	0.00001	0.00004	0	0.00206	0.00537
prop_mean	0.01113	0.00247	0.00082	0.0008	0.0001	0.00025	0	0.00077	0.00204

*Each value represents the mean variance of estimated property values of an particular object class consisting of 10 instances and their respective repetitions. Variances are scaled by color in ascending order from transparent (0) to red (highest variance)*.

The results of the (Table 2; Figure 9) reveal that the class variances are overall low, which implies stable property estimation methods in general. The highest variances can be found for the *flatness* property. The estimation of the *flatness* property for small and flat object instances is particularly affected by noise due to the low signal-to-noise ratio. Furthermore, it can be observed that for *ball, bowl*, and *to_go_cup* the variance of the *flatness* property is zero due to the fact that no top-level plane can be extracted for instances of these classes as they feature either round or negligible small top-level surfaces (see section 2.2.2). Similarly, a higher variance can be observed for the *rigidity* property which is caused by smaller object instances, such as *book, plate, sponge*, and *tray*. Here the detection of the first contact with the object causes false positives and therefore introduces varying deformation values.

In contrast, for the *hollowness* property the variance for *metal_box* and *sponge* are zero. Such object instances predominantly feature flat surfaces and negligible degree of hollowness. Considering sensor quantization effects, such negligible degree for hollowness cannot be confidently distinguished from sensor noise under such conditions (see section 2.2.3). As a consequence a default hollowness value of zero is set for instances that fall in a negligible range of hollowness, i.e., below 1 cm distance between marker. Concerning the *heaviness* property, a zero variance is observed due to the accurate measurement by a scale—considering a resolution of 1 g which is a sufficient resolution for our scenario.

#### 4.2.2. Property Coverage of RoCS

**Figure 10:**
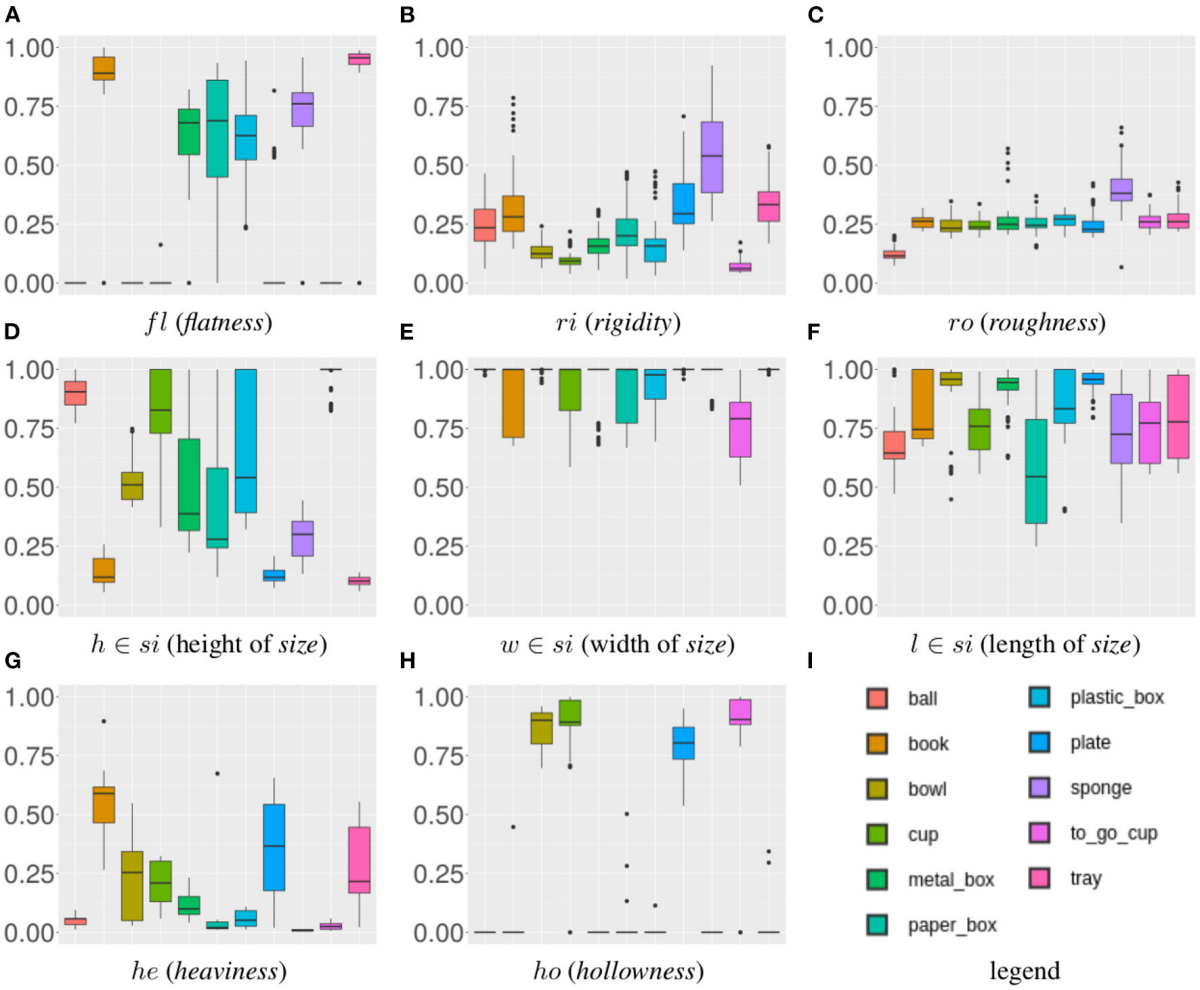
Category-wise coverage for each physical property [*fl, ri, ro, si, he, ho*].

The objective of this experiment is to evaluate the intra-class variance for each property in order to determine the range of data covered in each object class for one particular property. For this experiment, the mean estimated property value over the 10 repetitions is used. The result for each of the physical properties is shown in Figure 10 in form of a box plot in which all object instances of a particular class are considered.

Several observations can be made. For instance, *hollowness* and *flatness* are complementary in our dataset. Objects with *flatness* values close to zero are commonly exhibiting increased *hollowness* values (above 0.5) and vice versa. Only balls form an exception as they are neither flat nor hollow. While this means that we cover a wide range of values for the *flatness* property, we miss such coverage for *hollowness* values in the interval [0, 0.5]. Moreover, for *roughness* most object classes are in a similar range – except sponge and ball instances. As we place the objects in their most natural position we can conclude that the sponges' ground surfaces have a higher *roughness* due to their open-pored surfaces. Due to their roundish surfaces, ball instances feature obviously a low *roughness* value. Furthermore, it is unlikely to observe objects featuring *roughness* values close to one as none of the considered object classes has the ability to *stick* to the ramp.

For the *rigidity* values an interval of [0, 0.9] is covered, ranging from rigid objects such as *metal_box* to non-rigid objects such as *sponge*. Suspiciously, only a limited number of objects has a value of zero which indicates that sensor noise has its greatest effect on these objects.

Analyzing the *size* values, it becomes apparent that *width* commonly is the greatest dimension among the considered objects while the objects' height varies along the range of possible values.

#### 4.2.3. Property Correlation

In this experiment, we investigate the correlation in the physical properties of our data. Given estimated values of a particular property, we compute the mean property value $\bar{o_{x}}$ (Equation 11a) over the 10 repetitions for each object instance *o*. Based on these mean variances, the pearson correlation ρ_*XY*_ is obtained between two sets of mean variances *X* and *Y* corresponding to respective properties, see Equation (11b), where cov is the covariance and σ_*x*_ the standard deviation of *X*, respectively.

(11a) $$\begin{array}{ll} X & =\left\{\bar{o_{x_{1}}},\bar{o_{x_{2}}},\bar{o_{x_{3}}},...\right\} \end{array}$$

(11b) $$\begin{array}{ll} \rho_{XY} & =\frac{\text{cov}\left(X,Y\right)}{\sigma_{x}\sigma_{y}} \end{array}$$

Table 3 shows the pearson correlation among all physical properties with a color scale.

**Table 3:** Pearson Correlation on the mean values of physical properties.

	**Flatness**	**Rigidity**	**Roughness**	**s_length**	**s_width**	**s_height**	**Heaviness**
Flatness	–						
Rigidity	0.45	–					
Roughness	0.45	0.35	–				
size_length	0.03	0.12	0.15	–			
size_width	0.16	0.34	0.02	0.21	–		
size_height	-0.65	-0.59	-0.38	-0.26	-0.45	–	
Heaviness	0.09	-0.04	-0.13	0.19	0.02	-0.37	–
Hollowness	-0.71	-0.36	-0.08	0.24	-0.1	0.24	0.13

It can be observed that the correlation of our data is low in general. However, a strong negative correlation between *flatness* and *hollowness* is found which may indicate that in our data objects with high flatness are likely to have low hollowness. This matches our observation in section 4.2.2, where we noted the complementary nature of these properties in our dataset. The object instances of our dataset may also show some negative correlation between *size-height* and *flatness* as well as *size-height* and *rigidity*.

### 4.3. Property Semantics

Given a stable property estimation (section 4.2) from noisy real world data, the following experiment focuses on the semantic interpretation of the estimated object property values. We propose an experiment that groups object instances of our RoCS dataset in an unsupervised manner by considering a particular property or a set of properties. In order to conduct a preferably unbiased (machine-driven) grouping, *k*-means clustering is applied with a gradually increasing value of *k*={2, ..., 11}. Here, 11 is selected as upper bound as it represents the number of object classes considered in the RoCS dataset.

**Figure 11:**
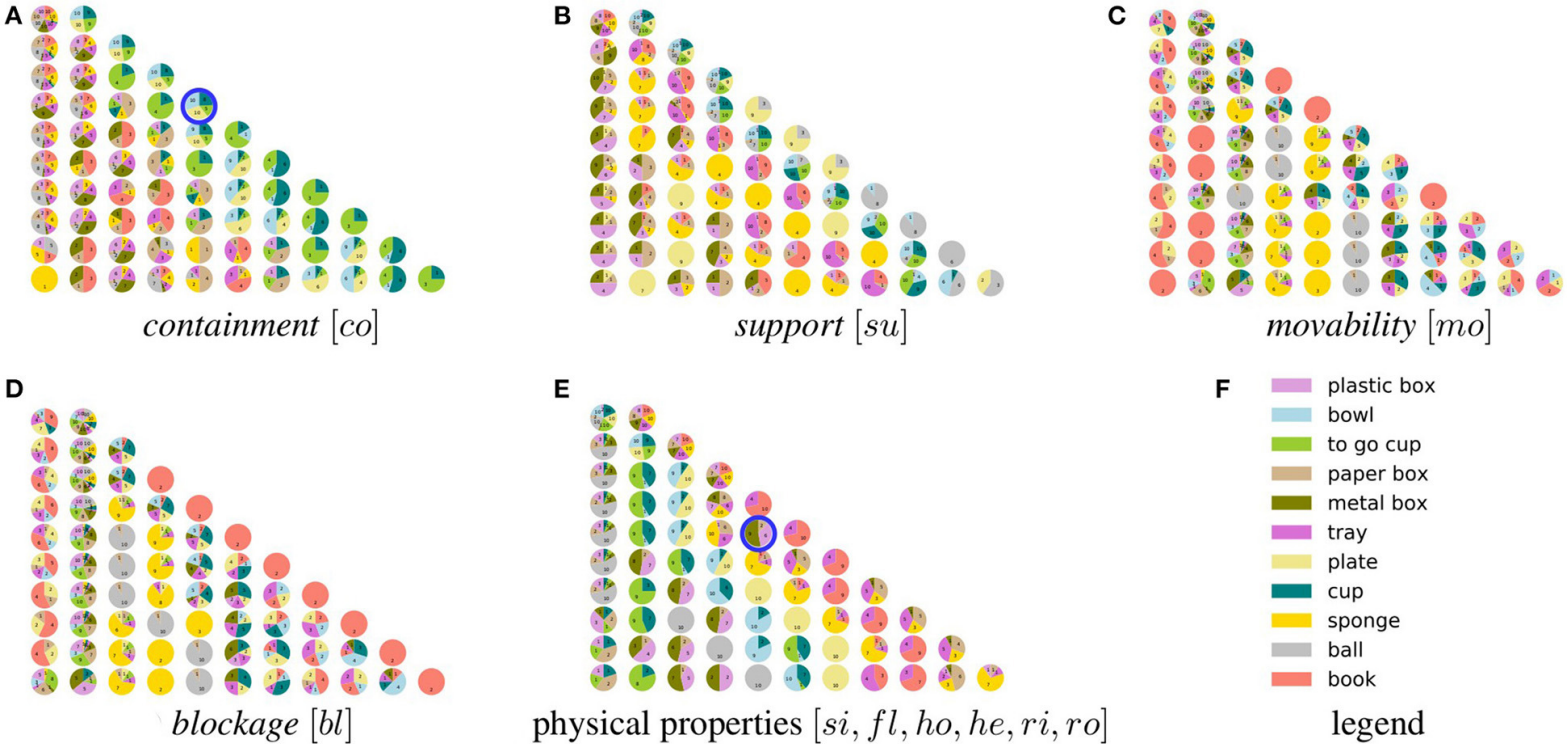
Gradual partitioning of instances to particular concepts given a particular set of properties describing each instance. Each concept is illustrated as a pie chart showing the object class label distribution of instances assigned to the respective concept. Sample concepts are annotated (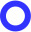) which illustrate object classes featuring similar quality regarding the property, such as *plate, bowl, cup, to_go_cup* regarding the *containment* property. One may observe, that given the assigned classes, object instances are not separable by the generated concepts. This is intended as it enables a quantitative analysis of similarity among instances and across classes based on the property.

Figure 11 consists of pyramid charts that shows the gradual partitioning process for the respective property. A group or a cluster is depicted as a pie-chart illustrating the distribution of assigned object instances with their labeled class. Therefore, each row of the pyramid-like structure shows the results of one application of the *k*-means clustering. The number of pie-charts in each row equals to number of clusters (*k* value).

Furthermore, since each group partitions the property space, assigned instances within the group share similar attributes. Therefore, a group can be interpreted as a *concept* representing a qualitative measurement of the respective property Generally on higher levels in the pyramids (lower *k*), the distribution of the instances and the classes in each concept is higher compared to lower levels (higher *k*). As a consequence, concepts in the higher levels appear more generic as opposed to the lower levels where concepts appear more specific.

Moreover, a pattern in the distribution of classes can be observed which is carried forward in the subsequent levels. This pattern hints toward semantic relations between class labels and observed concepts. For example, instances of *plate, bowl, cup, to_go_cup* share similar concepts regarding the *containment* property (see concept annotated with 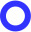 in Figure 11A) which is also reflected over multiple levels. Such patterns can also be observed and tracked over multiple levels for other functional properties in Figure 11. Furthermore, Figure 11E illustrates the gradual grouping process considering all physical properties of the object instances. Also here such patterns can be observed, e.g., on the right side where concepts have emerged that feature common properties related to instances such as *plastic_box, metal_box, paper_box* (see concept annotated with 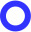 in Figure 11E).

As a result, the proposed property hierarchy (refer Figures 2B, 3B) allows to *discriminate* the object instances by associating them to meaningful groups featuring similar object concepts. In the figure, property generality can be observed across object classes, i.e., concepts on different granularity levels may feature dedications to instances of different object classes as they feature similar characteristics or trends regarding the property. This interrelation of object classes is reflected by the heterogeneity of the distribution of instances within a concept—even in case of *k*=11 when considering 11 object classes. These observations made in the proposed property acquisition procedure (Figure 7) provides a basis for the generation of conceptual knowledge about objects as shown in section 4.4.

### 4.4. Conceptual Knowledge for Substitute Selection

In this experiment, we demonstrate how the robot-centric conceptual knowledge grounded in the robot's sensory data can be successfully used to determine a substitute in a tool substitution scenario. While operating in a dynamic environment, a robot can not assume that a particular tool required in a task will always be available. In such scenarios, an ideal solution for a robot would be to improvise by finding a substitute for the missing tool as humans do.

To deal with such situation, we have developed an approach, called as ERSATZ (German word for a substitute) detailed in Thosar et al. (2018a), which is inspired by the way in which humans select a substitute in a non-invasive manner. In this approach, the robot-centric conceptual knowledge about objects is used to select a plausible substitute for a missing tool from the available objects. A tool, in this work, is defined as an artifact that is designed, manufactured, and maneuvered in accordance with its designated purpose in the task, such as hammer for hammering, tray for carrying, etc., and a substitute is seen as an alternate to a missing tool.

**Figure 12:**
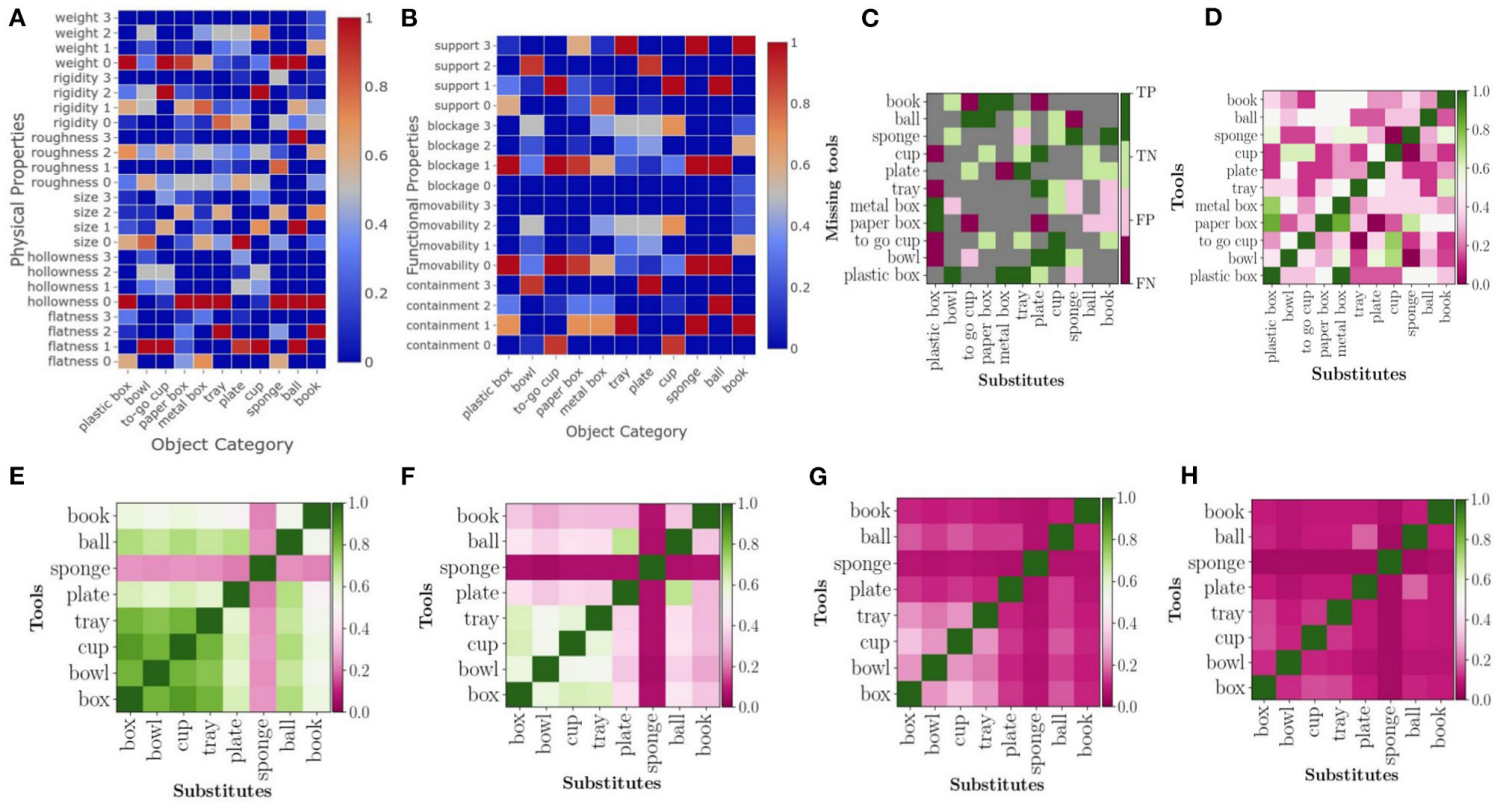
**(A)** Qualitative knowledge about physical properties; **(B)** Qualitative knowledge about functional properties;**(C)** The distribution of true positives (TP), true negatives (TN), false positives (FP), and false negatives (FN) in each substitution scenario; **(D)** Similarity between a tool and a substitute using Jaccard's Index; **(E)** Wu-Palmer similarity; **(F)** Lin Similarity; **(G)** Path similarity; **(H)** Jiang-Conrath Similarity.

For the experiments, we generated knowledge about 11 object classes using the approached discussed in the section 3. The dataset generated by RoCS was utilized for creating robot-centric conceptual knowledge about 11 object classes. The Figures 12A,B illustrates graphically the qualitative knowledge about physical and functional properties of 11 object classes as a heat map. The heat cells in the map represents the sample proportion of each qualitative measure in each object class. For instance, a single cell can be read as “the qualitative measure *Flatness 0* of the property class *Flatness* is *observed* in *60% instances* of class *Plastic box*”. Accordingly, in the figure, the conceptual knowledge suggests that all the instances of Plastic Box, Paper Box, Metal Box, Tray, Sponge, Ball, and Book are qualitatively similar with respect to the physical property Hollowness (i.e., none of them are hollow). The similar observation can be made for Tray, Sponge and Book with respect to the functional property Support (i.e., all of them may be stacked). Note that the indices assigned to each qualitative measure are not ordinal, but they are categorical. For instance, index 2 does not mean it is more valued than index 1. While comparative relationships exist in all four qualitative measurements of any property, they are not indicated by the given indices.

For the tool-substitution experiment, we generated 11 queries based on the 11 object classes, where each query consisted of a missing tool description and 5 randomly selected objects as available choices for a substitute. The queries were given to 21 human experts who were asked to select a substitute in each query. The expert selections were aggregated and selection proportion was calculated for each expert-selected substitute. ERSATZ used the knowledge generated in the previous section and computed substitute/s for each given query using the approach discussed in Thosar et al. (2020).

In order to validate a substitutability, the number of selected substitutes by human experts was then compared with the number of selected substitutes by ERSATZ. Similarly, the substitute selection by the 21 experts and ERSATZ in 11 scenarios are plotted as a heat map in Figure 12C. The grayed cells in the plots mean the corresponding object categories were not included in the available objects in the respective query. We used the conventional classification evaluation metrics: True Positives, True Negatives, False Positives, and False Negatives to evaluate the performance of ERSATZ. Our results showed that ERSATZ selected true positives in all 11 scenarios while true negatives in 8 scenarios. The results indicate that the proposed conceptual knowledge based substitute selection is meaningful and valid as confirmed by human selected substitutes. The experiment also demonstrates the successful application of the proposed conceptual knowledge, generated from the property data estimated from the proposed framework, in the tool substitution scenario.

#### 4.4.1. Robot-Centric Conceptual Knowledge vs WordNet

In this experiment, we have pitted our conceptual knowledge against WordNet in the substitute selection scenario. Our objective is to demonstrate that common sense knowledge bases such as WordNet are not adequate for substitute selection without selecting suitable knowledge a priori as done in Boteanu et al. (2016).

In this experiment, we compare the similarity among different objects determined by the similarity measures used in WordNet (Pedersen and Michelizzi, 2004) and Jaccard Index-based similarity proposed in Thosar et al. (2020). For the experiment, the path-length based measures *Path Similarity* and *Wu-Palmer Similarity* while information content based measures *Lin Similarity, Jiang-Conrath Similarity* were considered. We used the object labels from the multi-modal perception dataset, however some labels were adapted while using WordNet to compute the similarity. For instance, in the multi-modal data set we have a to-go-cup and a cup, however in WordNet there is no to-go-cup. Similarly, as WordNet does not differentiate between a plastic, a metal and a cardboard box, we considered only box for the WordNet comparison.

In Figures 12E–H, the resulting heat plot of the similarity between different objects using above mentioned similarity measures in WordNet is symmetric in nature while the relevant property driven Jaccard's Index based heat plot is asymmetric (see Figure 12D). This discrepancy is caused by the way objects are treated by WordNet and ERSATZ. While ERSATZ distinguishes between a tool and a substitute, WordNet does not make such a distinction. Therefore when the similarities between, say objects *A* and *B*, and between *B* and *A* are computed, the contents (path-length or information content) considered during the computation remain unchanged. Within the context of a specific designated purpose, the substitutability relationship between a tool and a substitute is symmetric, for instance, *for hammering*, a hammer can be replaced by a heeled shoe and vice versa. However, it is not the case once the context is shifted, for instance, a hammer can not be used as a heeled shoe for walking. Such an asymmetric relation is a necessity in tool substitution since it can not be assumed that if A is a substitute of a tool B, then B is a substitute of a tool A. Such assumption due to the symmetric relation may lead to an inadequate selection of a substitute as seen in the figure.

What the experiment shows us is that in order to use large knowledge bases such as WordNet or ConceptNet in substitute selection, simply applying similarity measures is not enough. Additionally these knowledge bases do not contain the exclusive information about physical and functional properties about objects. In contrast, our proposed knowledge generated from the quantitative measurements of physical and functional properties is desirable in substitute selection process as demonstrated in the above two experiments.

## 5. Conclusion

Retrieving conceptual knowledge about objects in the environment, in form of physical and functional properties, fundamentally contributes to an awareness about affordances provided by the environment to a robot. Such conceptual object knowledge is desired in various robotic scenarios (from household to industrial robotics) in order to efficiently perform tasks when dealing with objects in dynamic and uncertain environments. In scenarios where it is uncertain that a required tool is present, an efficient substitute selection is particularly required to successfully accomplish tasks. Besides substitute selection, conceptual object knowledge facilitates inferences about circumstances in situations in which the robot is applied to, e.g., if object *rigidity* is expected to be low, manipulation, including grasping strategies of the object, can be accordingly adapted to increase a successful object handling.

However, state-of-the-art conceptual knowledge approaches are generally hand-crafted and generated from a human perspective in form of natural language concepts and may not be suitable for substitute selection as demonstrated in the experiment in section 4.4.1. Consequently, the discrepancy between human and robotic capabilities (e.g., visual, auditory, haptic perception, prior knowledge, etc.) is also reflected in the knowledge generation process conducted by humans and the then necessarily complex interpretation for it by robotic systems. In order to mitigate this discrepancy, we proposed a robot-centric approach as we believe that conceptual knowledge has to be generated considering the individual robotic capabilities, so to say in form of robotic language concepts.

A multi-modal approach for object property estimation and generation of robot-centric knowledge has been proposed to acquire conceptual knowledge from a robotic perspective. We introduced a bottom-up knowledge acquisition process, from capturing sensory data over a numeric estimation of object properties, to a symbolic conceptualization of objects' properties. Experiments have revealed the *stability* as well as the *inter-class generality* of the proposed object property acquisition procedure. This outcome provides a basis for the subsequent conceptual knowledge generation in the context of the *substitute selection* scenario. Tool substitution results have demonstrated the applicability of the generated conceptual knowledge.

We conclude, that the proposed robot-centric and multi-modal conceptualization approach may contribute to equip a robot with the capability to reason about objects on a conceptual level compared to general approaches which are only based on e.g., visual (image pixels) or haptic (resistance feedback) sensory data. Moreover, such robot-centric and multi-modal knowledge can be applied to a variety of scenarios beyond substitute selection. To facilitate further use, we established the RoCS dataset and made it publicly available.

As the goal of this work was a robot-centric conceptual knowledge generation, our future work is directed, toward the *transfer* of such knowledge among heterogeneous robotic systems. To facilitate the development and collaborative progress in this framework, we aim to develop an online system where developers can plug-in their estimation methods (simple or more complex) for the same property or new property to the framework requiring minimal or more sophisticated experimental set-ups. This way, we wish to create a community of users who can select the estimation methods based on the sensor and robot availability at their end.

## Data Availability Statement

The original contributions presented in the study are publicly available. This data can be found here: https://gitlab.com/rocs_data/rocs-dataset.

## Author Contributions

MT primarily contributed to the conception and preliminary design of the robot-centric knowledge acquisition framework. MT, CM, and GJ contributed to the property selections and definitions of RoCS framework. CM and GJ contributed to the implementation of the framework and extraction methods. NP, RM, and SJ contributed to the dataset generation of 110 objects. CM, GJ, and JS contributed in the integration and the evaluation of the dataset. MT, CM, GJ, and MP contributed to evaluation of the property semantics. MT contributed to the conceptual knowledge generation and application of the conceptual knowledge in tool substitution scenario. AB, MP, and SZ contributed to the critical evaluation of the work. All authors contributed to manuscript revision, read, and approved the submitted version.

## Conflict of Interest

The authors declare that the research was conducted in the absence of any commercial or financial relationships that could be construed as a potential conflict of interest.

## References

[B1] Abelha P. Guerin F. (2017). “Learning how a tool affords by simulating 3D models from the web,” in IEEE International Conference on Intelligent Robots and Systems (Vancouver), 4923–4929.

[B2] Abelha P. Guerin F. Schoeler M. (2016). “A model-based approach to finding substitute tools in 3D vision data,” in Proceedings - IEEE International Conference on Robotics and Automation (Stockholm).

[B3] Agostini A. Aein M. J. Szedmak S. Aksoy E. E. Piater J. Worgotter F. (2015). “Using structural bootstrapping for object substitution in robotic executions of human-like manipulation tasks,” in IEEE International Conference on Intelligent Robots and Systems (Hamburg), 6479–6486.

[B4] Awaad I. Kraetzschmar G. K. Hertzberg J. (2014). “Challenges in finding ways to get the job done,” in Planning and Robotics (PlanRob) Workshop at 24th International Conference on Automated Planning and Scheduling (Portsmouth).

[B5] Baber C. (2003). Cognition and Tool Use London: Taylor and Francis.

[B6] Bansal R. Tuli S. Paul R. Mausam (2020). TOOLNET: using commonsense generalization for predicting tool use for robot plan synthesis. arXiv preprint arXiv:2006.05478.

[B7] Biro D. Haslam M. Rutz C. (2013). Tool use as adaptation. Philos. Trans. R. Soc. Lond. B. Biol. Sci. 368:20120408. 10.1098/rstb.2012.0408 PMC4027410 24101619

[B8] Bischoff R. Huggenberger U. Prassler E. (2011). “Kuka youbot - a mobile manipulator for research and education,” in 2011 IEEE International Conference on Robotics and Automation (Shanghai), 1–4.

[B9] Boesch C. (2013). “Chap. 2: Ecology and cognition of tool use in chimpanzees,” in Tool Use in Animals: Cognition and Ecology, eds Sanz J. B. C. Call C. M. (Cambridge: Cambridge University Press), 21–47.

[B10] Boteanu A. St. Clair A. Mohseni-Kabir A. Saldanha C. Chernova S. (2016). Leveraging large-scale semantic networks for adaptive robot task learning and execution. Big Data 4, 217–235. 10.1089/big.2016.0038 27992263

[B11] Brown S. Sammut C. (2012). “A relational approach to tool-use learning in robots,” Inductive Logic Programming - 22nd International Conference (Dubrovnik), 1–15.

[B12] Brown S. Sammut C. (2013). “A relational approach to tool-use learning in robots,” Lecture Notes in Computer Science (including subseries Lecture Notes in Artificial Intelligence and Lecture Notes in Bioinformatics) Berlin: Springer 7842 LNAI, 1–15.

[B13] Coradeschi S. Saffiotti A. (2003). An introduction to the anchoring problem. Robot. Auton. Syst. 43, 85–96. 10.1016/S0921-8890(03)00021-6

[B14] Daoutis M. Coradeshi S. Loutfi A. (2009). Grounding commonsense knowledge in intelligent systems. J. Ambient Intell. Smart Environ. 1, 311–321. 10.3233/AIS-2009-0040

[B15] Emery N. J. (2013). “Chap. 4: Insight, imagination and invention: tool understanding in a non-tool-using corvid,” in Tool Use in Animals: Cognition and Ecology, eds Sanz C. M. Call J. Boesch C. (Cambridge: Cambridge University Press), 67–88.

[B16] Feldman J. Narayanan S. (2004). Embodied meaning in a neural theory of language. Brain Lang. 89, 385–392. 10.1016/S0093-934X(03)00355-9 15068922

[B17] Fellbaum C. (ed.). (1998). WordNet: An Electronic Lexical Database. Cambridge, MA ; London: The MIT Press.

[B18] Fischler M. A. Bolles R. C. (1981). Random sample consensus: a paradigm for model fitting with applications to image analysis and automated cartography. Commun. ACM 24, 381–395. 10.1145/358669.358692

[B19] Gallese V. Lakoff G. (2005). The brain's concepts: the role of the sensory-motor system in conceptual knowledge. Cogn. Neuropsychol. 22, 455–479. 10.1080/02643290442000310 21038261

[B20] Gärdenfors P. (2007). “Cognitive semantics and image schemas with embodied forces,” in Embodiment in Cognition and Culture, eds Krois J. M. Rosengren M. Stedele A. Westerkamp D. (John Benjamins Publishing Company), 57–76. Available online at: https://portal.research.lu.se/portal/en/publications/cognitive-semantics-and-image-schemas-with-embodied-forces(336b0bea-162b-4acb-8f8e-62cfb006f05a).html#Overview

[B21] Garrido-Jurado S. Munoz-Salinas R. Madrid-Cuevas F. Marin-Jimenez M. (2014). Automatic generation and detection of highly reliable fiducial markers under occlusion. Pattern Recogn. 47, 2280–2292. 10.1016/j.patcog.2014.01.005

[B22] Gavin R Hunt A. T. Gray R. (2013). “ Chap. 5: Why is tool use rare in animals?,” in Tool Use in Animals: Cognition and Ecology, eds Sanz J. B. C. Call C. M. (Cambridge: Cambridge University Press), 89–118.

[B23] Gibson J. J. (1986). “Chap. 8: The theory of affordances,” in The Ecological Approach to Visual Perception (New York, NY: Psychology Press; Taylor & Francis Group), 127–143.

[B24] Gupta R. Kochenderfer M. J. (2004). “Common sense data acquisition for indoor mobile robots,” in Proceedings of the Nineteenth National Conference on Artificial Intelligence, Sixteenth Conference on Innovative Applications of Artificial Intelligence (San Jose, CA), 605–610.

[B25] Harnad S. (1990). The symbol grounding problem. Physica D 42, 335–346. 10.1016/0167-2789(90)90087-6

[B26] Hartson R. (2003). Cognitive, physical, sensory, and functional affordances in interaction design. Behav. Inform. Technol. 22, 315–338. 10.1080/01449290310001592587

[B27] Hernik M. Csibra G. (2009). Functional understanding facilitates learning about tools in human children. Curr. Opin. Neurobiol. 19, 34–38. 10.1016/j.conb.2009.05.003 19477630

[B28] Hodges J. R. Spatt J. Patterson K. (1999). “What” and “how”: evidence for the dissociation of object knowledge and mechanical problem-solving skills in the human brain. Proc. Natl. Acad. Sci. U.S.A. 96, 9444–9448. 10.1073/pnas.96.16.9444 10430962 PMC17802

[B29] Kaboli M. Feng D. Cheng G. (2017). Active tactile transfer learning for object discrimination in an unstructured environment using multimodal robotic skin. Int. J. Humanoid Robot. 15:1850001. 10.1142/S0219843618500019

[B30] Kim J. Lim H. Ahn S. C. Lee S. (2018). “RGBD camera based material recognition via surface roughness estimation,” in Proceedings - 2018 IEEE Winter Conference on Applications of Computer Vision, WACV 2018, Stateline.

[B31] Koubâa A. (2017). Robot Operating System (ros): The Complete Reference, Vol. 2. Cham: Springer.

[B32] Kraft D. Pugeault N. Baseski E. Popovic M. Kragic D. Kalkan S.. (2009). Erratum: “Birth of the object: detection of objectness and extraction of object shape through object–action complexes”. Int. J. Humanoid Robot. 6:561. 10.1142/S0219843609001772

[B33] Kuhn W. (2007). “An image-schematic account of spatial categories,” in Spatial Information Theory (Berlin: Springer), 152–168.

[B34] Lemaignan S. Ros R. Mösenlechner L. Alami R. Beetz M. (2010). “ORO, a knowledge management platform for cognitive architectures in robotics,” in IEEE/RSJ 2010 International Conference on Intelligent Robots and Systems, IROS 2010 - Conference Proceedings (Taipei), 3548–3553.

[B35] Lenat D. B. (1995). Cyc: a large-scale investment in knowledge infrastructure. Commun. ACM 38, 33–38. 10.1145/219717.219745

[B36] Li W. Fritz M. (2015). “Teaching robots the use of human tools from demonstration with non-dexterous end-effectors,” in IEEE-RAS International Conference on Humanoid Robots (Seoul), 547–553.

[B37] Lim G. H. Suh I. H. Suh H. (2011). Ontology-based unified robot knowledge for service robots in indoor environments. IEEE Trans. Syst. Man and Cybern. Part A Syst. Hum. 41, 492–509. 10.1109/TSMCA.2010.2076404

[B38] Liu H. Singh P. (2004). ConceptNet – A practical commonsense reasoning tool-kit. BT Technol. J. 22, 211–226. 10.1023/B:BTTJ.0000047600.45421.6d

[B39] Lloyd S. (1982). Least squares quantization in pcm. IEEE Trans. Inform. Theor. 28, 129–137. 10.1109/TIT.1982.1056489

[B40] Louwerse M. M. Jeuniaux P. (2010). The linguistic and embodied nature of conceptual processing. Cognition 114, 96–104. 10.1016/j.cognition.2009.09.002 19818435

[B41] Mustafa W. Wächter M. Szedmak S. Agostini A. Kraft D. Asfour T.. (2016). “Affordance estimation for vision-based object replacement on a humanoid robot,” in 47th International Symposium on Robotics Munich. Vol. 2016, 164–172.

[B42] Osiurak F. Rossetti Y. Badets A. (2017). What is an affordance? 40 years later. Neurosci. Biobehav. Rev. 77, 403–417. 10.1016/j.neubiorev.2017.04.014 28432011

[B43] Pedersen T. Michelizzi J. (2004). “WordNet :: similarity - measuring the relatedness of concepts measures of relatedness,” in Nineteenth National Conference on Artificial Intelligence (AAAI-04) (San Jose, CA), 1024–1025.

[B44] Pineda L. A. Rodríguez A. Fuentes G. Rascón C. Meza I. (2017). A light non-monotonic knowledge-base for service robots. Intell. Serv. Robot. 10, 159–171. 10.1007/s11370-017-0216-y

[B45] Rand A. (1990). “Chap. 2: Concept-formation,” in Introduction to Objectivist Epistemology, eds Binswanger H. Peikoff L. (New York, NY: Plume Books) 25–43.

[B46] Sanz C. M. Call J. Boesch C. (eds.). (2013). Tool Use in Animals: Cognition and Ecology. Cambridge: Cambridge University Press.

[B47] Saxena A. Jain A. Sener O. Jami A. Misra D. K. Koppula H. S. (2014). RoboBrain: large-scale knowledge engine for robots. arXiv preprint arXiv:1412.0691.

[B48] Shrivatsav N. Nair L. Chernova S. (2019). Tool substitution with shape and material reasoning using dual neural networks. arXiv preprint arXiv:1911.04521.

[B49] Sinapov J. Wiemer M. Stoytchev A. (2009). Interactive learning of the acoustic properties of household objects. in IEEE International Conference on Robotics and Automation Kobe.

[B50] Spiers A. J. Liarokapis M. V. Calli B. Dollar A. M. (2016). Single-grasp object classification and feature extraction with simple robot hands and tactile sensors. IEEE Trans. Haptics 9, 207–220. 10.1109/TOH.2016.2521378 26829804

[B51] Stoytchev A. (2007). Robot tool behavior: a developmental approach to autonomous tool use (Ph.D. dissertation), College of Computing, Georgia Institute of Technology, 1–277.

[B52] Stückler J. Behnke S. (2014). “Adaptive tool-use strategies for anthropomorphic service robots,” in 14th IEEE-RAS International Conference on Humanoid Robots (Humanoids) Madrid.

[B53] Suh I. H. Lim G. H. Hwang W. Suh H. Choi J. H. Park Y. T. (2007). “Ontology-based multi-layered robot knowledge framework (OMRKF) for robot intelligence,” in IEEE International Conference on Intelligent Robots and Systems (San Diego, CA), 429–436.

[B54] Susi T. Ziemke T. (2005). “On the subject of objects: four views on object perception and tool use,” Cogn. Commun. Cooperat. 3, 6–19. 10.31269/triplec.v3i2.19

[B55] Swoboda D. M. (2014). A comprehensive characterization of the asus xtion pro depth sensor. Jr. J. Sci. Technol. 1, 16–20.

[B56] Takahashi K. Ogata T. Tjandra H. (2014). “Tool – body assimilation model based on body babbling and neuro-dynamical system,” in International Conference on Artificial Neural Networks (Hamburg).

[B57] Takamuku S. Gómez G. Hosoda K. Pfeifer R. (2007). “Haptic discrimination of material properties by a robotic hand,” in 2007 IEEE 6th International Conference on Development and Learning, ICDL (London).

[B58] Tenorth M. Beetz M. (2009). “KNOWROB- Knowledge processing for autonomous personal robots,” in IEEE/RSJ International Conference on Intelligent Robots and Systems (Missouri), 4261–4266.

[B59] Thosar M. Mueller C. Zug S. (2018a). “What stands-in for a missing tool?: A prototypical grounded knowledge-based approach to tool substitution,” in 11th International Workshop on Cognitive Robotics in 16th International Conference on Principles of Knowledge Representation and Reasoning (Tempe, AZ).

[B60] Thosar M. Mueller C. A. Jäger G. Pfingsthorn M. Beetz M. Zug S.. (2020). “Substitute selection for a missing tool using robot-centric conceptual knowledge of objects,” in Knowledge Representation and Reasoning Track in 35th ACM/SIGAPP Symposium On Applied Computing (Brno).

[B61] Thosar M. Zug S. Skaria A. M. Jain A. (2018b). “A review of knowledge bases for service robots in household environments,” in 6th International Workshop on Artificial Intelligence and Cognition (Palermo).

[B62] Tikhanoff V. Pattacini U. Natale L. Metta G. (2015). “Exploring affordances and tool use on the iCub,” in IEEE-RAS International Conference on Humanoid Robots (Seoul), 130–137.

[B63] Toussaint M. Allen K. R. Smith K. A. Tenenbaum J. B. (2019). “Differentiable physics and stable modes for tool-use and manipulation planning - Extended abstract,” in IJCAI International Joint Conference on Artificial Intelligence (Macao), 6231–6235.

[B64] Vaesen K. (2012). The cognitive bases of human tool use. Behav. Brain Sci. 35, 203–218. 10.1017/S0140525X11001452 22697258

[B65] Vauclair J. Anderson J. A. (1994). Object manipulation, tool use, and the social context in human and non-human primates. Techniq. Cult. 23–24, 121–136. 10.4000/tc.556

[B66] Wicaksono H. Sammut C. (2018). Tool use learning for a real robot. Int. J. Electr. Comput. Eng. 8, 1230–1237. 10.11591/ijece.v8i2.pp1230-1237

[B67] Wu J. Lim J. J. Zhang H. Tenenbaum J. B. Freeman W. T. (2016). “Learning physical object properties from unlabeled videos,” in British Machine Vision Conference (York).

[B68] Xie A. Ebert F. Levine S. Finn C. (2019). Improvisation through physical understanding: using novel objects as tools with visual foresight. arXiv preprint arXiv:1904.05538.

[B69] Zhu Y. Fathi A. Fei-Fei L. (2014). “Reasoning about object affordance in a knowledge based representation,” European Conference on Computer Vision (Amsterdam), 408–424.

